# Root metabolite profiles support a chemical-trophic filtering hypothesis for genotype- and stage-specific rhizosphere assembly in chicory

**DOI:** 10.3389/fmicb.2026.1855632

**Published:** 2026-07-17

**Authors:** Lalie Leclercq, Gaëtan Kientz, Emily Lloret, Cécile Palaric, Bernard Taminiau, Georges Daube, Roland Molinié, Jean-Xavier Fontaine, David Mathiron, Djamel Drider, Ali Siah, Bruno Desprez, Marine Cordonnier, Jean-Louis Hilbert, Anca Lucau-Danila

**Affiliations:** 1 UMRt 1158 BioEcoAgro, Univ. Lille, JUNIA, Univ. Liège, UPJV, Univ. Artois, ULCO, INRAE, Lille, France; 2 Joint Laboratory CHIC41H Univ. Lille-Florimond Desprez, Cité scientifique, Villeneuve d’Ascq, France; 3 ULR 4515 - LGCgE, Laboratoire de Génie Civil et géo-Environnement, Univ. Lille, Lille, France; 4 Department of Food Sciences, Microbiology, FARAH, Univ. Liege, Liege, Belgium; 5 Plateforme Analytique UFR des Sciences, UPJV, Amiens, France; 6 Florimond Desprez Veuve and Fils, Cappelle-en-Pévèle, France

**Keywords:** chemical-trophic filtering, *Cichorium intybus*, plant genotype, rhizosphere microbiome, root metabolites

## Abstract

The rhizosphere is a dynamic interface where plant- and soil-derived factors jointly influence microbial community assembly. In chicory (*Cichorium intybus* L.), the respective roles of genotype, developmental stage, and root metabolites in structuring rhizosphere communities remain insufficiently understood. This study aimed to characterize patterns of microbial assembly and to assess their associations with root metabolite profiles. Rhizosphere and bulk soil from three chicory genotypes were sampled at two developmental stages and analyzed using bacterial and fungal metabarcoding. Diversity metrics, differential abundance analyses, and literature-based functional annotation were integrated with root metabolite profiling to explore associations between microbial taxa and metabolite profiles. Rhizosphere microbial communities associated with chicory differed from bulk soil and were structured by plant genotype and developmental stage. Lower α-diversity at early stages may reflect the selective enrichment of specific taxa, suggesting non-random assembly. Community variation was associated with root metabolite profiles, including primary metabolites and sesquiterpene lactones (STLs). Across development, the microbiome shifted from taxa linked to nutrient transformation and microbial interactions including *Nitratireductor*, *Sphingomonas*, and *Serratia*, toward communities dominated by saprotrophic and organic matter-degrading taxa such as *Streptomyces*, *Pseudarthrobacter*, and *Lecanicillium*. Genotype-dependent differences further suggested that plant genetic background contributed to rhizosphere assembly patterns. However, these relationships are correlative, and the underlying mechanisms require validation through targeted experimental approaches. The observed correlations led us to propose a hypothesis of temporally structured chemical-trophic filtering, meaning that plant metabolites and soil nutrient conditions jointly contribute to shaping microbial communities in a genotype-dependent manner.

## 1 Introduction

Industrial chicory (*Cichorium intybus* var. *sativum*) is a major crop cultivated worldwide for applications in human nutrition and animal feed. Chicory is also recognized as a functional food due to its high inulin content, a well-established prebiotic that promotes beneficial gut microbiota (Gibson et al., 2017), as well as its richness in specialized secondary metabolites such as chlorogenic acids and sesquiterpene lactones (STLs). These compounds exhibit documented antimicrobial and allelopathic properties (Chadwick et al., 2013; Pouille et al., 2022). Industrial chicory represents a particularly relevant model for investigating plant–soil–microbe interactions due to its agronomic importance, high production of bioactive STLs, and well-documented hardiness and resilience across diverse environmental conditions (Hilbert and Rambaud, 2023; Leclercq et al., 2025). Microbial communities inhabiting the rhizosphere are shaped by multiple interacting factors, including soil properties, environmental conditions, agricultural practices, and plant developmental stage (Philippot et al., 2013; Hartman et al., 2017). Host genotype, root physiology, and metabolism may also influence microbial recruitment and contribute to selective community assembly from the surrounding soil reservoir (Bulgarelli et al., 2013; Schlaeppi et al., 2014; Tkacz et al., 2015; Zhalnina et al., 2018). In this context, the rhizosphere constitutes a dynamic interface where plant roots actively modulate microbial assembly through the release of primary and secondary metabolites, suggesting that plant-derived compounds may act as ecological filters shaping microbial colonization (Bais et al., 2006; Badri and Vivanco, 2009).

Root-derived primary metabolites are known to influence rhizosphere microbial communities by providing carbon and energy sources for soil microorganisms. Soluble sugars, organic acids, and amino acids constitute major components of root exudates and can contribute to plant–microbe interactions in the rhizosphere (Badri and Vivanco, 2009; Dennis et al., 2010; Jones et al., 2009; Philippot et al., 2013; Sasse et al., 2018).

Beyond primary metabolites, plant secondary metabolites contribute to a more selective shaping of rhizosphere microbial communities, acting as chemical signals or inhibitors that influence microbial assembly. Among these secondary metabolites, STLs are recognized as highly bioactive compounds that may contribute to the selective shaping of rhizosphere microbial communities. STLs, including those produced by *Asteraceae* such as chicory, are well known for their antimicrobial properties, although their effects are strongly dose-dependent and species-specific (Häkkinen et al., 2021). Their activity is largely attributed to the presence of an α-methylene-γ-lactone moiety capable of reacting with nucleophilic thiol groups in proteins via Michael-type addition, leading to covalent alkylation of cysteine residues and disruption of essential enzymatic and redox-regulated cellular processes (Schmidt, 2006; Chadwick et al., 2013). Gram-positive bacteria are often reported to be more susceptible to STLs, likely due to the absence of an outer membrane barrier and the increased accessibility of intracellular targets (Picman, 1986). In fungi, similar thiol-reactive mechanisms can interfere with key metabolic enzymes and membrane-associated functions, resulting in growth inhibition or altered physiological activity (Picman, 1986; Chadwick et al., 2013). Importantly, such effects rarely result in complete microbial elimination but instead generate selective pressure that differentially affects sensitive taxa (Badri and Vivanco, 2009; Bais et al., 2006; Inderjit and Duke, 2003).

In crops producing bioactive secondary metabolites, genotype-dependent variation in root physiology, metabolism, and exudation patterns may strongly influence microbial recruitment and rhizosphere ecological structuring by shaping the selection of microorganisms from the surrounding soil reservoir (Pascale et al., 2020; Trivedi et al., 2020). The concept of chemical-trophic filtering provides a useful framework for interpreting plant-associated microbiome assembly, whereby microbial communities may be influenced by both chemical selection pressures arising from plant-derived compounds and trophic constraints related to nutrient availability (Philippot et al., 2013; Pascale et al., 2020; Trivedi et al., 2020). However, the mechanisms and ecological strategies by which genotype-specific metabolite profiles shape bacterial and fungal community assembly in industrial chicory, particularly across developmental stages, remain insufficiently understood. The objective of this study was therefore to determine assess the influence of genotype and developmental stage on rhizosphere bacterial and fungal communities in industrial chicory and to assess whether variation in root metabolite composition is associated with differential microbial recruitment patterns. To achieve a comprehensive characterization of the root microbiota and root metabolome, multiple analytical platforms were employed, including microbial DNA sequencing, NMR for global metabolic profiling, and UHPLC–HRMS for sensitive detection of specialized metabolites. By integrating metabolomic and metabarcoding approaches, we aimed to characterize genotype- and stage-dependent patterns of rhizosphere microbial assembly and to evaluate their associations with root metabolite profiles in the chicory rhizosphere.

## 2 Materials and methods

### 2.1 Plants and sampling

Seeds of three industrial chicory genotypes (*Cichorium intybus* var. *sativum*), Far01, Far05, and Far10, were provided by Florimond Desprez Veuve and Fils (Cappelle-en-Pévèle, France). The seeds were cultivated in the same experimental field located in Coutiches, northern France (50°27’56.9”N 3°13’04.4”E). The area is characterized by a temperate oceanic climate, with a mean annual temperature of 12.2 °C and an annual precipitation of 492 mm in 2022 (Météo-France data).^1^ The experiment was conducted during the 2022 growing season. Wheat was cultivated on the plot during the previous growing season, followed by a fallow period before chicory sowing. Standard agronomic practices included soil tillage prior to sowing, nitrogen fertilization with ammonium nitrate in March 2022, foliar fertilization in June 2022, and conventional weed and disease management throughout the growing season. No irrigation was applied during the experiment. The complete crop management protocol is described in Supplementary File 1.

The field experiment followed a randomized plot design to minimize spatial heterogeneity within the experimental area. Each genotype was represented by 7 independent plots distributed across the field, with 10 rows per plot. To avoid edge effects, plants were sampled from the central rows of each plot. At each developmental stage, healthy plants were randomly selected throughout the plot area.

For soil characterization, five topsoil samples (0–15 cm depth) were collected in May 2022 using a grid-based random sampling method. Sampling points were selected using random coordinates within the grid, with a minimum distance of 10 m between points to ensure representative field coverage. Approximately 500 g of soil was collected at each location using sterile tools and transferred to clean, non-reactive polyethylene bags without additives. Samples were stored in a cool, dry environment until analysis.

Two plant developmental stages were investigated. The first harvest took place in July 2022 (T1), corresponding to the young plant stage with a fine taproot of approximately 10–15 cm, and the second harvest in September 2022 (T2), corresponding to the mature stage characterized by a tuberized root of approximately 30–35 cm. Because T1 and T2 samples were collected at different periods of the growing season, developmental stage effects could not be fully separated from seasonal and environmental variation.

At each stage, five plants per genotype were randomly selected and carefully uprooted. After gently shaking off loose soil, the roots were collected. Soil adhering to the roots was gently scraped off and considered rhizosphere soil (Rhi). Control bulk soil samples (Ctrl) were collected from unvegetated areas located approximately 2 m away from chicory plants, at a depth corresponding to the chicory root zone (approximately 10–20 cm), although residual rhizosphere influence within the cultivated field cannot be completely excluded. Five independent bulk soil samples were obtained. All Rhi and Ctrl samples were placed into sterile 50 mL Falcon tubes, freeze-dried, ground, homogenized, and stored until DNA extraction. Roots were cleaned to remove soil and debris, rinsed with tap water to remove adhering soil particles, followed by a rinse with distilled water, and then freeze-dried prior to metabolite analysis.

### 2.2 Soil analysis

Physicochemical properties of bulk soil were determined using subsamples allocated to different analyses. Specifically, 20 g of soil were used for the determination of water pH (pH_*water*_), 50 mg for total carbon (TC), total nitrogen (TN) and total organic carbon (TOC) analyses, and 5 g for major element analysis. All measurements were performed in triplicate. For pH_*water*_ determination, soil samples were manually ground and sieved at 2 mm. The < 2 mm fraction was mixed with deionized water at a soil:solution ratio of 1:2.5 (FAO, 2021) and stirred for 30 min. After decantation, pH was measured using a Hanna probe (HI 2211), with a precision of 0.05 pH unit.

For the remaining analyses, soil samples were finely ground to a particle size of < 10 μm. TC and TN concentrations were determined at the Laboratoire d’Océanologie et de Géosciences, Lille, France using an elemental analyzer (ThermoFisher Flash EA 1112 Series). TOC was measured using the same instrument following sample decarbonation with diluted hydrochloric acid (HCl, 1:5). TOC content was also calculated as the difference between total carbon and inorganic carbon, the latter quantified as CaCO_3_ by calcimetry using a Bernard calcimeter. Analytical precision for TC, TN, and TOC measurements was 0.05 %. The TOC/TN ratio was subsequently calculated. The elemental analyses for major elements were conducted at the SARM (Service d’Analyse des Roches et des Minéraux, Nancy, France) using an ICP-OES (Inductively Coupled Plasma-Optical Emission Spectrometry, iCap6500). Detection limits for SiO_2_, Al_2_O_3_, Fe_2_O_3_, MnO, MgO, CaO, Na_2_O, K_2_O, TiO_2_, and P_2_O_5_ were 0.050, 0.040, 0.015, 0.015, 0.030, 0.030, 0.020, 0.030, 0.020, and 0.100%, respectively. The loss on ignition (LOI) was also determined and corresponds to the mass difference between the sample mass before and after calcination.

### 2.3 DNA extraction and amplicon sequencing

Total DNA from rhizosphere, control soil or roots was extracted from 5 samples per experimental condition according to NucleoSpin DNA Soil Kit (Macherey-Nagel, Düren, Germany). DNA amounts were quantified by using BioSpectrometer (Eppendorf, Hamburg, Germany) and DNA quality was assessed with the 2100 Bioanalyzer (Agilent, Santa Clara, United States). Following DNA extraction and quality assessment, the three samples per condition showing the highest DNA quality and amplification efficiency were selected (Supplementary File 2) to ensure sufficient sequencing quality and coverage, and were subsequently sent for sequencing to the GIGA sequencing platform (Liège University, Belgium). Although this selection strategy was implemented to ensure robust sequencing quality, the reduced number of biological replicates (*n* = 3) may limit statistical power and should therefore be considered when interpreting downstream analyses. For bacterial DNA sequencing the amplification of the V1-V2 region of the 16S rDNA and the library preparation were performed with the following primers: direct (5’-GAGAGTTTGATYMTGGCTCAG-3’) and reverse (5’-ACCGCGGCTGCTGGCAC-3’). For fungal DNA sequencing, the amplification of the Internal Transcribed Spacer (ITS) region 5.8S-ITS2, and the library were prepared for each sample using universal primers with Illumina overhang adapters targeting the ITS2 region. The forward primer (5’-GCATCGATGAAGAACGCAGC-3’), and the reverse primer (5’-TCCTCCGCTTATTGATATGC-3’) were used for their broad coverage of fungal taxa. Single-organism DNA (*Escherichia coli* for bacteria and *Saccharomyces cerevisiae* for fungi) was used as a positive control, while a no-template control was used as a negative control to confirm the PCR amplifications. No detectable amplification or evidence of significant sequencing contamination was observed in the negative controls. Each PCR product was purified with the Agencourt AMPure XP Beads Kit (Beckman Coulter, Pasadena, United States), then subjected to a second PCR round for indexing, using Nextera XT index 1 and 2 primers. Following purification, PCR products were quantified using Quant-iT PicoGreen (Thermo-Fisher Scientific, Waltham, United States) and diluted to 10 ng⋅μL^–1^. Final quantification of each library sample was performed with the KAPA SYBR FAST (KapaBiosystems, Wilmington, United States) before standardization, pooling, and sequencing on a MiSeq sequencer with v3 reagents (Illumina, San Diego, United States). Data processing involved the mothur v1.44 package and the VSEARCH algorithm (Rognes et al., 2016) for alignment, clustering, and chimera detection as described by Gérard et al. (2021). The mean number of cleaned reads before subsampling was 108,017 reads per sample for ITS2 and 89,427 reads per sample for 16S datasets. After filtering and chimera removal, 90.6% of ITS2 reads and 78.3% of 16S reads were retained. Sequences were next clustered into operational taxonomic units (OTUs) at 97% identity using the bioinformatics workflow initially implemented for this study (Leclercq et al., 2025). Alignment and taxonomic identification were carried out using the SILVA database (release 132) for bacterial 16S rRNA gene sequences and the UNITE database for fungal ITS sequences (5.8S rDNA region). Rarefaction to 10,000 reads per sample was performed to standardize sequencing depth across samples before downstream metabarcoding analyses. High Good’s coverage values at the OTU level (99.4% for ITS2 and 98.7% for 16S) indicated that the sequencing depth was sufficient to capture the vast majority of the diversity present in the samples. OTUs were subsequently aggregated into phylotypes at the phylum and genus taxonomic levels. All raw reads from biosamples were deposited at the National Center for Biotechnology Information (NCBI) under BioProject accession number PRJNA1441179.

### 2.4 Metabarcoding analysis

Based on the rarefied table of 10,000 reads per sample, rare OTUs representing less than 0.01% of total reads across all samples were filtered out prior to diversity analyses to reduce the influence of sequencing artifacts and spurious low-abundance taxa. Filtered data were examined for alpha diversity using three indices: Shannon’s diversity (Shannon, 1948), Pielou’s evenness (Pielou, 1966), and Chao1 richness (Chao, 1984). Alpha diversity indices were calculated using “phyloseq” package in R. Statistical differences were analyzed using the rank-based Aligned Rank Transform (ART) procedure implemented in the “ARTool” package, followed by factorial ANOVA including Genotype, Stage, and their interaction as fixed factors. In addition, bulk soil controls were compared with rhizosphere samples from each genotype at each developmental stage using one-factor ART analyses. Pairwise post-hoc comparisons were performed using estimated marginal means with the “emmeans” package and adjusted for multiple testing using Tukey’s procedure (*p <* 0.05 was considered significant). GraphPad Prism 9.0 (GraphPad Software, San Diego, CA, United States) was used to generate graphical representations.

Filtered data were subsequently used for β-diversity analyses to evaluate dissimilarities in microbial community composition among samples. Community dissimilarities were calculated using Bray–Curtis distances (Bray and Curtis, 1957). Differences in microbial community structure were tested using permutational multivariate analysis of variance (PERMANOVA; Anderson, 2001) implemented in the *adonis2()* function (999 permutations). The effects of genotype, developmental stage, and their interaction were assessed on rhizosphere samples, whereas rhizosphere and bulk soil communities were compared separately. Homogeneity of multivariate dispersions was verified using the *betadisper()* function. All analyses were performed in R (v4.5.2) using the “vegan” package (Oksanen et al., 2022). Community patterns were visualized by principal coordinates analysis (PCoA).

Filtered data were also used for relative abundance analyses following total sum scaling normalization. Differential abundance analyses were conducted separately at three taxonomic levels (phylum, genus, and species), for two developmental stages (T1 and T2), and for bacteria and fungi independently. Differentially abundant taxa were identified using DESeq2 (Love et al., 2014). Raw count data were modeled using a negative binomial generalized linear model, with genotype or developmental stage included as fixed effects depending on the comparison. Size factors were estimated using the poscounts method, which is robust to the high sparsity and zero inflation typical of microbiome datasets. *P*-values were adjusted for multiple testing using the Benjamini–Hochberg false discovery rate (FDR; Benjamini and Hochberg, 1995), and taxa with adjusted *p* < 0.05 were considered significant. For graphical representation, average relative abundances were calculated for each condition, and figures were generated using GraphPad Prism 9 (GraphPad Software, San Diego, CA, United States).

### 2.5 Functionnal characterization

Functional characterization was performed using publicly available reference genomes representative of the identified taxa and annotated through the RAST server,^2^ BacDive^3^ and literature for bacterial species. Specifically, bacterial species were retained based on a mean relative abundance threshold > 1% across samples in order to focus on dominant rhizosphere members. For each retained species, one representative reference genome was manually downloaded from the NCBI Assembly database (“reference genome” category) and submitted to the RAST server for annotation using the SEED subsystems framework. The RAST server was used to explore the presence of genes associated with phosphorus (P), iron (Fe), nitrogen (N), potassium (K), and sulfur (S) metabolism, as well as genes related to virulence/defense mechanisms and phytohormone biosynthesis. FungiDB ^4^ and literature data completed functions for fungal species.

### 2.6 Root metabolite extraction

Roots were freeze-dried for 48 h and, depending on the sampling stage, either the entire root system (T1) or homogenized root subsamples (T2, due to increased root biomass) were ground into a fine powder using a ball mill for 30 s at 30 Hz. Fifty-four milligrams of powder were extracted with 1.8 mL of a methanol/water mixture (25:75, v/v). The mixture was shaken at 950 rpm for 20 min at 20 °C using a ThermoMixer^®^ (Eppendorf AG, Hamburg, Germany), followed by centrifugation at 13,400 rpm for 10 min at 4 °C (Centrifuge 5810 R, Eppendorf AG, Hamburg, Germany). The supernatant was then filtered through a 0.22 μm PVDF membrane. Crude extracts were stored at –20 °C until further analyses.

### 2.7 Metabolite analysis by NMR

An aliquot of 800 μL of crude extract supernatant were freeze-dried and reconstituted in 800 μL of a methanol-d4/KH_2_PO_4_ buffer (0.1 M) in D_2_O (50:50, v/v), adjusted to pH 6.0, and containing TMSP (0.0125%), NaN3 (0.6 mg⋅mL^−1^), and maleic acid (1 mM). Samples were vortexed, sonicated for 10 min, and transferred to 5 mm NMR tubes. Analyses were performed on 5 biological replicates per genotype at each developmental stage, except for Far05 at T1 (*n* = 4 due to insufficient sample quantity).

NMR spectra were acquired at 300 K on an Avance III 600 MHz spectrometer (600.13 MHz for ^1^H; Bruker BioSpin, Wissembourg, France) equipped with a 5 mm TXI inverse probe with z-gradient. Spectra were processed using TopSpin v3.6.2 (Bruker). Standard processing procedures, including apodization, zero-filling, phase correction, and baseline correction, were applied. Acquisition parameters were selected according to Pouille et al. (2022). Briefly, ^1^H NMR spectra were acquired using the noesypr pulse sequence with 64 scans and a spectral width of 8403 Hz.

Two-dimensional NMR experiments were also performed. J-resolved (J-res) spectra were acquired using the jresesgp pulse sequence with 16 scans and spectral widths of 8417 Hz in the F1 dimension and 50 Hz in the F2 dimension. Two-dimensional heteronuclear single quantum coherence (HSQC) spectra were acquired using the hsqcetgpprsisp pulse sequence with 16 scans and spectral widths of 8417.5 Hz in F2 and 26,412 Hz in F1.

For NMR spectra, a pre-processing step was performed using NMRProcFlow software (v.1.4) to align spectra with a maximum relative shift of 0.05 ppm, remove background noise and methanol/water signals, exclude the 3–5 ppm region due to extensive signal overlap associated with inulin, and perform spectral binning using the “smart binning” mode to avoid splitting multiplets and prevent potential redundancy errors. A resolution factor of 0.5 and a signal-to-noise ratio (SNR) threshold of 3 were applied. A matrix of 324 variables was then generated for subsequent statistical analyses. To compare annotated compounds levels in Far01, Far05, and Far10 at T1 and T2 stages, statistical analyses were performed using the Kruskal–Wallis test. When significant differences were detected, Bonferroni-corrected *post hoc* pairwise comparisons were conducted. Statistical significance was set at *p* < 0.05.

### 2.8 Metabolite analysis by UHPLC–HRMS

Crude extracts were diluted 20-fold in LC–MS grade methanol/water (20:80, v/v) and filtered through a 0.22 μm PVDF membrane. UHPLC–HRMS analyses were performed using an ACQUITY UPLC I-Class system coupled to a Vion IMS QTOF mass spectrometer (Ion Mobility Quadrupole Time-of-Flight) equipped with an electrospray ionization (ESI) source (Waters, Manchester, United Kingdom).

One microliter of each sample was injected, and chromatographic separation was achieved on a Kinetex Biphenyl column (100 × 2.1 mm, 1.7 μm; Phenomenex, Torrance, CA, United States) maintained at 55 °C. The mobile phase consisted of water containing 0.1% formic acid (A) and methanol containing 0.1% formic acid (B), delivered at a flow rate of 0.55 mL⋅min^−1^. The gradient program was as follows (A:B): 80:20 (0 min), 80:20 (0.5 min), 40:60 (5 min), 10:90 (6 min), 10:90 (7 min), 80:20 (7.5 min), and 80:20 (10 min).

The ESI source was operated in both positive and negative ionization modes with a capillary voltage of 2.5 kV and a cone voltage of 20 V. Source and desolvation temperatures were set at 120 and 450 °C, respectively. Nitrogen was used as both the desolvation and drying gas at flow rates of 800 and 50 L⋅h^−1^, respectively. For accurate mass measurements, a leucine enkephalin solution (m/z 554.2615) was continuously infused as a lock-mass reference.

The TOF analyzer was operated in sensitivity mode, providing an average mass resolution of approximately 50,000 (FWHM). HDMS^E^ spectra were acquired in continuum mode over a mass range of m/z 50–2,000 with a scan time of 0.2 s. Two independent scans with alternating collision energies were recorded during each acquisition cycle: a low-energy (LE) scan at 6 eV and a high-energy (HE) scan with collision energies ramped from 10 to 15 eV. Data acquisition and processing were performed using UNIFI software (v1.9.4, Waters).

From the UHPLC–HRMS analytical data, an initial matrix of 1,344 variables was generated. After data processing and quality filtering, a final dtaset of 148 variables was retained. Each variable was retained in the matrix if it met the following criteria: a coefficient of variation (CV) below 35%, an intensity at least 10 times higher than the blank, and presence in all replicates of the same condition.

To identify variables contributing to sample discrimination, HRMS^E^ spectra were analyzed. This data-independent acquisition (DIA) method, combined with ion mobility, allows programmed fragmentation during sample elution by alternating collision energies without prior selection of precursor ions. As a result, two spectra are acquired within a single run: a low-energy (LE) spectrum providing information on intact molecular ions, and a high-energy (HE) spectrum containing fragment ion information. This acquisition mode enables the collection of accurate mass and fragmentation data in a single analysis. Electrospray ionization spectra acquired in both negative (ESI-) and positive (ESI+) modes were used for marker annotation. Detected ions were characterized by their mass-to-charge ratio (m/z) as [M - H]^–^ or [M + H]^+^ ions, respectively. In addition, the formation of common adducts during ionization was considered, including [M + HCOO]^–^, [M + HSO_4_]^–^, [M + Na]^+^, and [M + NH_4_]^+^. Metabolite annotation was performed based on the comparison of chromatographic retention times and mass spectra, calculation of putative molecular formulas from accurate precursor ion masses, and analysis of fragment ions.

Principal component analyses (PCA) were performed using R software (v. 4.3.0) with the “FactoMineR” package. Heatmaps were generated using GraphPad Prism 9 (GraphPad Software, San Diego, CA, United States).

### 2.9 Correlation analysis

Correlation analyses between genotype-specific metabolites and rhizosphere taxonomic composition were performed using R (v4.5.2). Associations were evaluated between signature microbial taxa (bacteria and fungi) identified as differentially abundant in the rhizosphere and eleven specialized metabolites detected in chicory roots. Microbial relative abundance data were transformed using centered log-ratio (CLR) transformation to account for compositionality, whereas metabolite peak intensities were log_2_-transformed to stabilize variance. Regularized canonical correlation analysis (rCCA) was conducted using the “mixOmics” R package to identify multivariate correlations between microbial genera and metabolite profiles. In addition, Spearman’s rank correlation coefficients were computed separately for each developmental stage to assess pairwise associations. To control for multiple testing, *p*-values were adjusted using the Benjamini–Hochberg false discovery rate (FDR) procedure. Correlations with adjusted *p* < 0.05 were considered statistically significant.

## 3 Results

### 3.1 Soil analysis

According to the “Référentiels Régionaux Pédologiques” (IGCS-RPP, GIS Sol, n.d.; Baize and Girard, 2009) and the soil map available on the Geoportail website,^5^ the soil type for the experimental plot was classified as Brunisol-Redoxisol (IUSS WRB, 2022).

Mean values of pH_*water*_, as well as concentrations of total carbon (TC), total nitrogen (TN), total organic carbon (TOC), major elements, and loss on ignition (LOI), are presented in Table 1. The mean pH_*water*_ of the bulk soil was 7.04, indicating neutral conditions. This value is consistent with those typically reported for Brunisols and Redoxisols developed on calcareous or chalky parent materials, where soil pH generally remains below 7.5 under cultivated conditions (Baize and Girard, 2009). Total nitrogen (TN), total carbon (TC), and total organic carbon (TOC) contents averaged 0.15 ± 0.02%, 2.56 ± 0.10%, and 2.22 ± 0.10%, respectively. TOC values suggest a moderate organic matter content typical of cultivated soils, whereas TN levels indicate relatively low nitrogen availability. The relatively narrow difference between TC and TOC suggests a limited contribution of inorganic carbon, consistent with the low CaO content (1.15 ± 0.45%). The resulting TOC/TN ratio (∼14.8) falls within the upper range commonly reported for cropland soils (8–15), reflecting balanced organic matter turnover and moderate nitrogen availability (Kirkby et al., 2011; Cotrufo et al., 2021). Loss on ignition (LOI) averaged 7.38 ± 0.46%, supporting the interpretation of moderate organic matter content. This value is consistent with LOI ranges reported for cultivated mineral soils, where organic matter levels are generally reduced compared to natural ecosystems due to enhanced mineralization and biomass export (Heiri et al., 2001).

**TABLE 1:** Chemical properties and major element composition of the experimental soil.

Parameter	Mean ± SD
pHwater	7.04 ± 0.14
TN (%)	0.15 ± 0.02
TC (%)	2.56 ± 0.1
TOC (%)	2.22 ± 0.1
SiO_2_ (%)	78.30 ± 0.72
Al_2_O_3_ (%)	6.85 ± 0.09
Fe_2_O_3_ (%)	2.83 ± 0.06
MnO (%)	0.049 ± 0.003
MgO (%)	0.51 ± 0.01
CaO (%)	1.15 ± 0.45
Na_2_O (%)	0.71 ± 0.01
K_2_O (%)	1.66 ± 0.02
TiO_2_ (%)	0.63 ± 0.01
P_2_O_5_ (%)	0.23 ± 0.06
LOI (%)	7.38 ± 0.46

Values are means ± standard deviation (*n* = 3). Concentrations are expressed as % (w/w) for TN, TC, and TOC, and in wt% oxides for major elements.

Major element composition was dominated by SiO_2_ (78.30 ± 0.72%), and aluminum and iron oxides (Al_2_O_3_: 6.85 ± 0.09%; Fe_2_O_3_: 2.83 ± 0.06%) were present in moderate amounts, consistent with the presence of aluminosilicate minerals such as clays and feldspars. Minor oxides, including K_2_O (1.66 ± 0.02%) and Na_2_O (0.71 ± 0.01%), further support the contribution of feldspathic minerals, while MgO and MnO occurred at low concentrations, as typically observed in temperate agricultural soils (Kabata-Pendias, 2011). The relatively low CaO content suggests limited carbonate abundance, in agreement with the measured TOC and near-neutral pH.

Overall, the physicochemical characteristics of the soil are representative of a moderately fertile, mineral-dominated agricultural soil with active carbon and nitrogen cycling.

### 3.2 Microbial diversity

**FIGURE 1:**
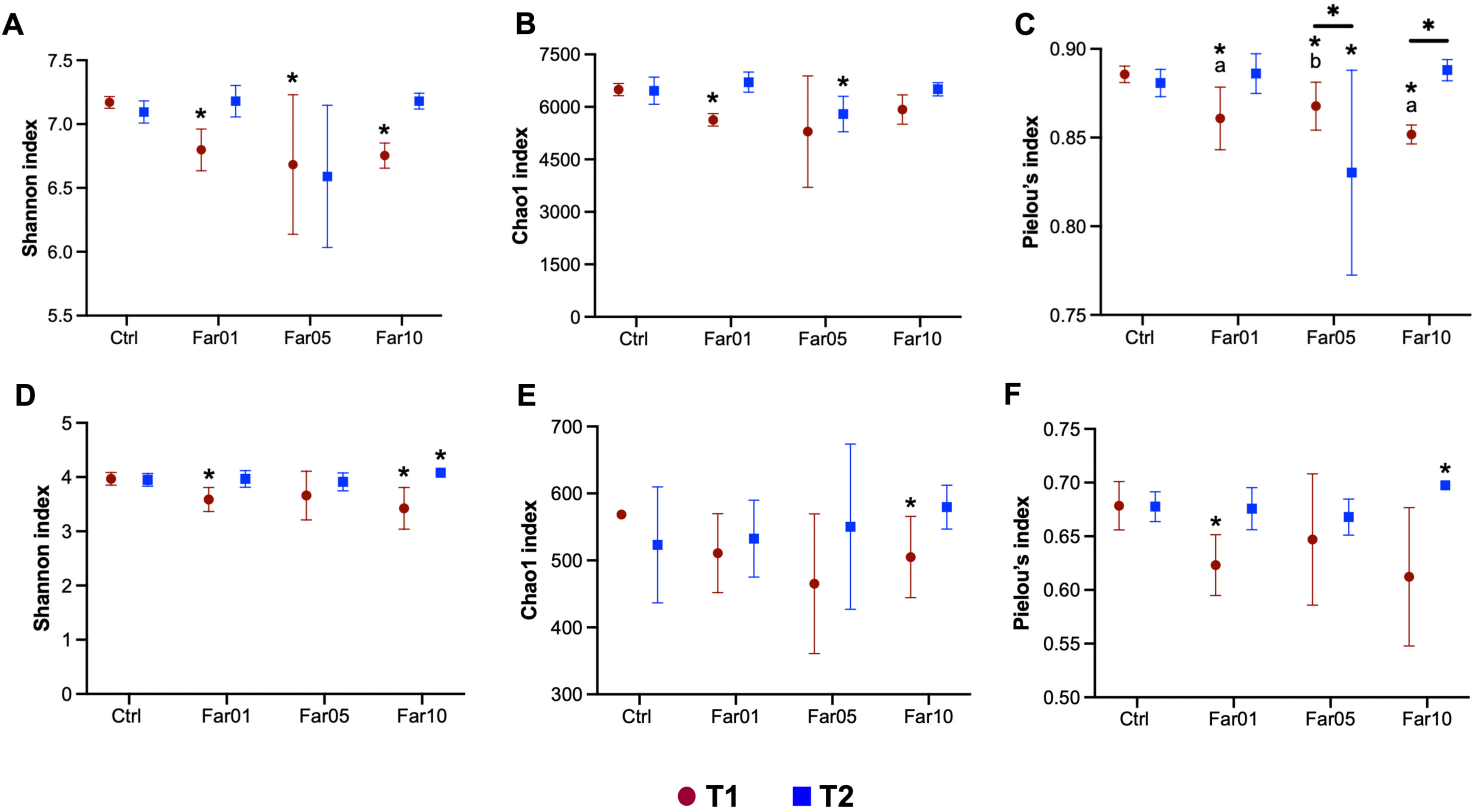
Diversity, richness and evenness of bacterial **(A–C)** and fungal **(D–F)** communities associated with Far01, Far05, and Far10 chicory genotypes. Shannon-Weaver diversity, Chao1 index and Pielou’s evenness were computed with R functions from the “phyloseq” package. An asterisk above the data point indicates significant differences between rhizosphere and control soil. Different letters indicate significant differences among genotypes at a given stage. Significant differences between T1 and T2 for the same genotype are indicated by a bar with an asterisk (ART ANOVA, Tukey’s test *p* < 0.05).

To evaluate the microbial diversity, evenness and richness of different chicory genotype we observed the alpha diversity illustrated by Shannon, Pielou’s and Chao1 indices (Figure 1).

**FIGURE 2:**
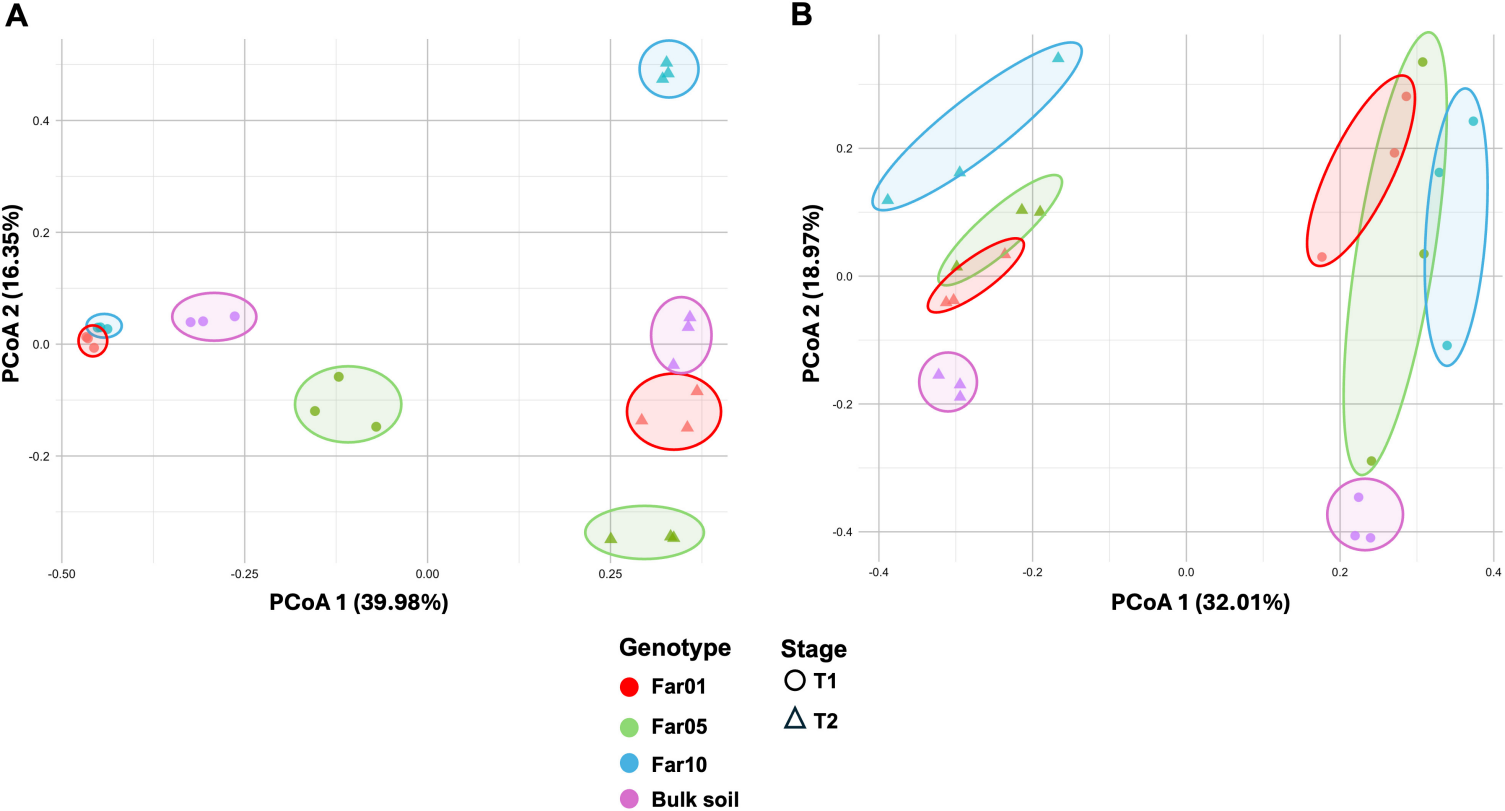
Principal coordinate analysis (PCoA) representing beta-diversity for bacteria **(A)** and fungi **(B)**. Bulk soil and rhizosphere taxa were represented for each genotype (Far01, Far05, and Far10) and each stage of development (T1 and T2).

In terms of diversity (Shannon index), samples collected at the T1 developmental stage exhibited a significantly lower microbial diversity for all genotypes (*p* ≤ 0.02) compared with the bulk soil control, with a more pronounced effect observed for bacterial communities. Shannon index values ranged from 6.68 to 6.80 in the rhizosphere compared with 7.17 in the control for bacteria, and from 3.43 to 3.66 compared with 3.97 for fungi (Figures 1A,D). Microbial richness, estimated using the Chao1 index, was also reduced at T1 relative to the control, with values ranging from 5294 to 5926 in the rhizosphere compared with 6,494 in the control for bacteria, and from 465 to 510 compared with 568 for fungi. This decrease was statistically significant for the bacterial community associated with Far01 (*p* = 0.02) and for the fungal community associated with Far10 (*p* = 0.02) (Figures 1B,E). Similarly, community evenness assessed using Pielou’s index showed a consistent reduction at T1 for both bacterial and fungal communities, ranging from 0.85 and 0.86 in the rhizosphere compared with 0.88 in the control for bacteria, and from 0.61 to 0.64 compared with 0.67 for fungi. These differences reached statistical significance for bacterial communities across all genotypes (*p* = 0.02) and for fungal communities associated with Far01 (*p* = 0.02) (Figures 1C,F). At T1, a significant difference in bacterial evenness was also observed among the three genotypes, particularly between Far01 and Far10 (*p* = 0.03) and between Far05 and Far10 (*p* = 0.01) (Figure 1C). Overall, these patterns suggest a stronger selective recruitment of bacterial and fungal communities at the early developmental stage (T1), whereas fewer significant changes relative to the control or among genotypes were detected at T2. Microbial beta-diversity, reflecting differences in community composition based on OTU distribution, was visualized using principal coordinates analysis (PCoA) (Figure 2). Distinct bacterial and fungal community structures were observed according to both genotype and developmental stage. These patterns were supported by PERMANOVA analyses. For bacteria, significant effects of genotype (*R*^2^ = 28.1%, *p* = 0.001), developmental stage (*R*^2^ = 37.1%, *p* = 0.001), and their interaction (*R*^2^ = 23.9%, *p* = 0.001) were detected (Supplementary File 3). Similarly, for fungi, genotype (*R*^2^ = 20.1%, *p* = 0.001), developmental stage (*R*^2^ = 34.4%, *p* = 0.001), and their interaction (*R*^2^ = 18.3%, *p* = 0.001) significantly shaped community composition (Supplementary File 3).

### 3.3 Microbial abundance

**FIGURE 3:**
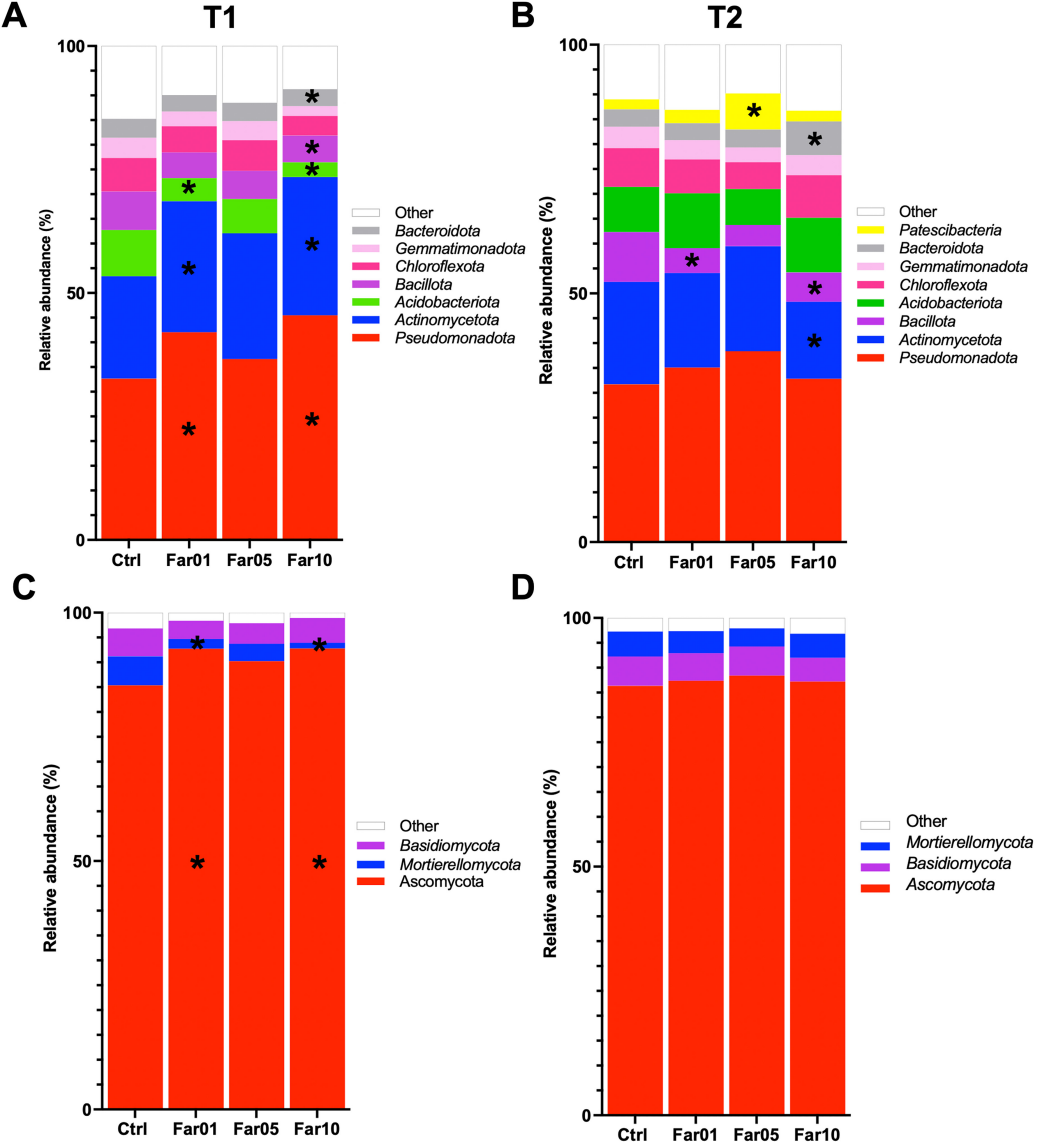
Relative abundance of microbial phyla in bulk soil (Ctrl) and rhizosphere samples for three genotypes (Far01, Far05, and Far10) at two developmental stages (T1 and T2). Bacterial phyla were shown for T1 **(A)** and T2 **(B)**, and fungal phyla for T1 **(C)** and T2 **(D)**. Only taxa with a relative abundance ≥ 3% were shown individually, while taxa below this threshold were grouped under “Other.” Asterisks indicate significant differences compared to the control (*p* < 0.05; DESeq2).

**FIGURE 4:**
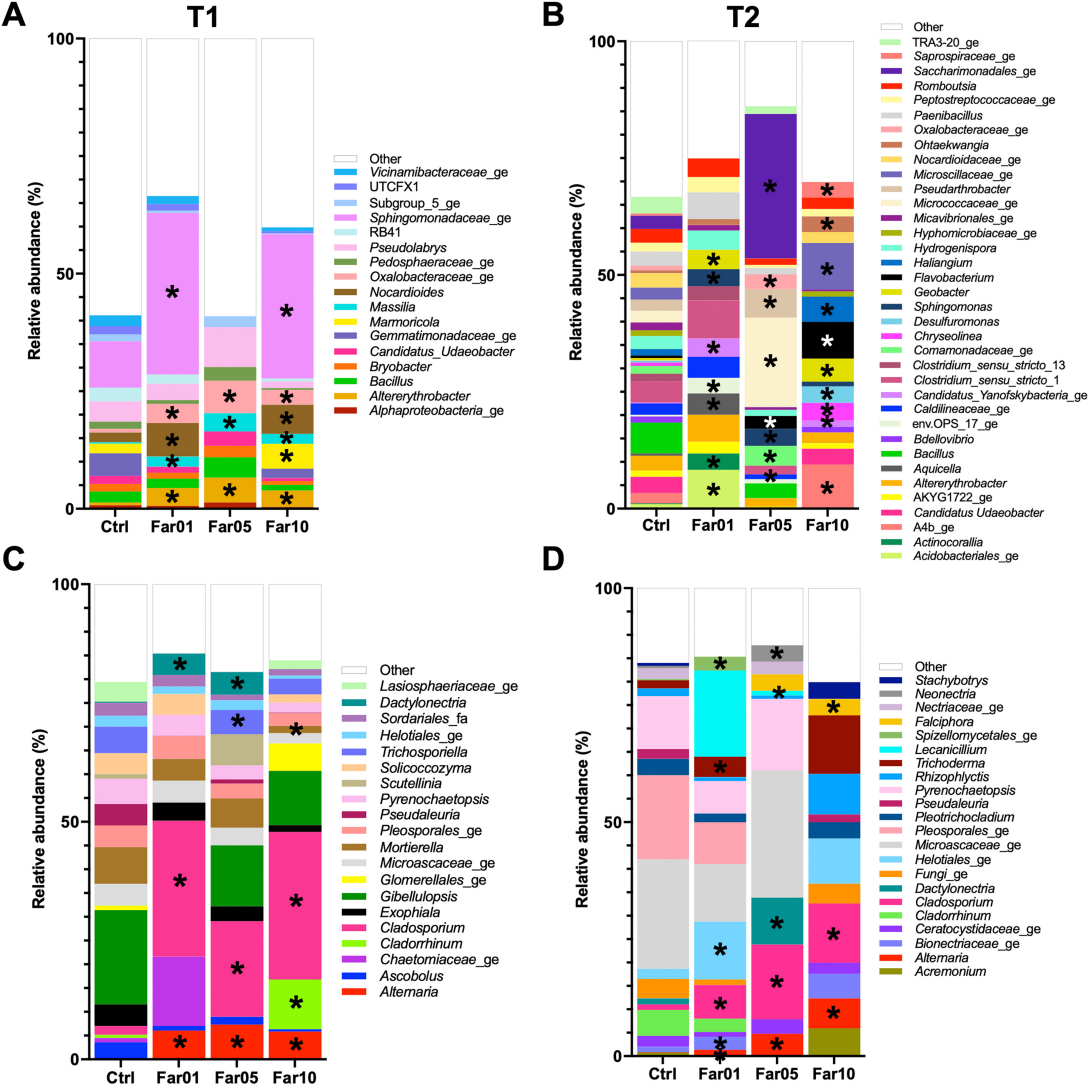
Relative abundance of microbial genera in bulk soil (Ctrl) and rhizosphere samples for three genotypes (Far01, Far05, and Far10) at two developmental stages (T1 and T2). Bacterial genera are shown for T1 **(A)** and T2 **(B)**, and fungal genera for T1 **(C)** and T2 **(D)**. Only taxa with a relative abundance ≥ 3% are shown individually, while taxa below this threshold were grouped under “Other.” Asterisks indicate significant differences compared to the control (*p* < 0.05; DESeq2).

**FIGURE 5:**
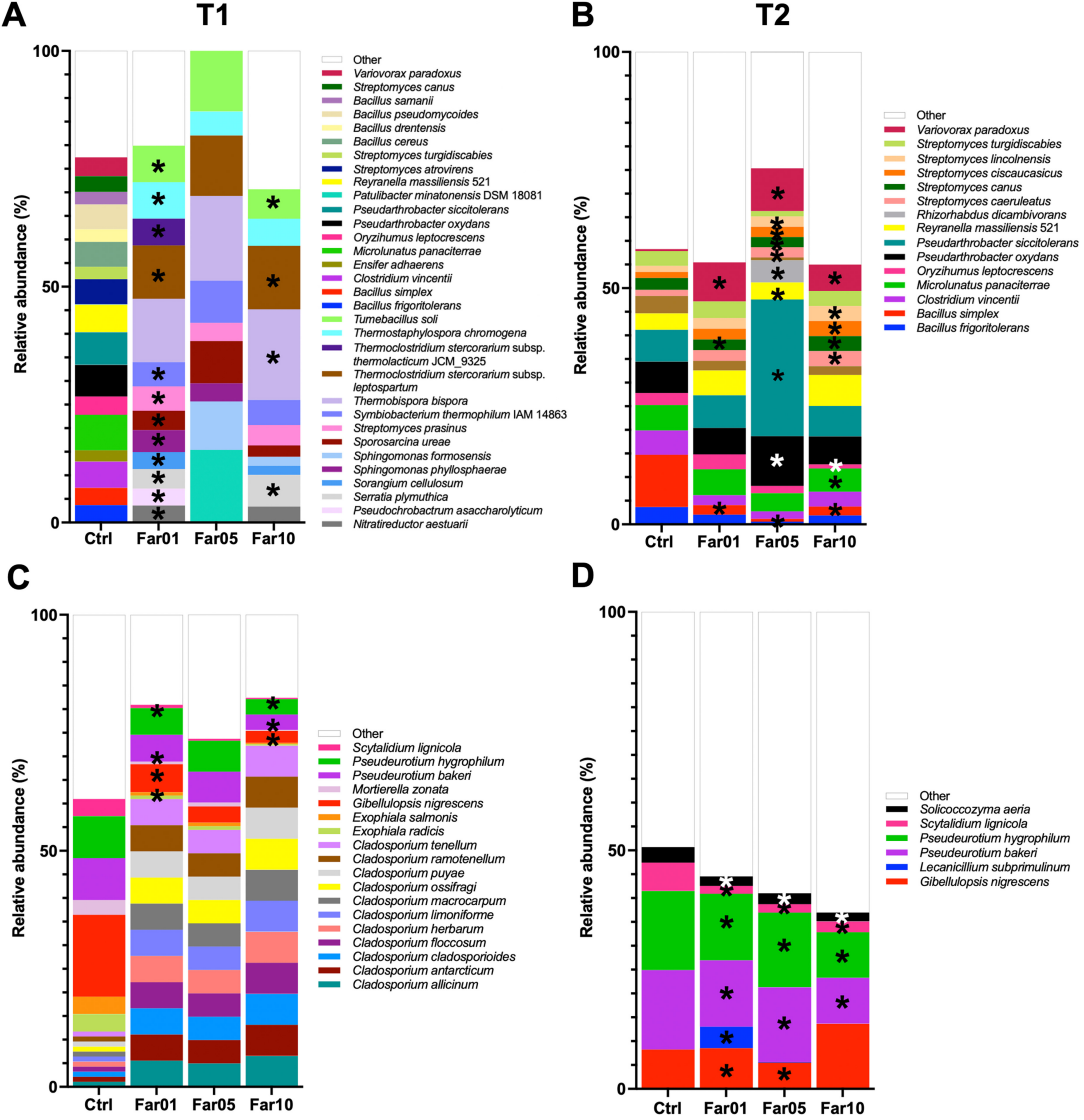
Relative abundance of microbial species in bulk soil (Ctrl) and rhizosphere samples for three genotypes (Far01, Far05, and Far10) at two developmental stages (T1 and T2). Bacterial species are shown for T1 **(A)** and T2 **(B)**, and fungal species for T1 **(C)** and T2 **(D)**. Only taxa with a relative abundance ≥ 3% are shown individually, while taxa below this threshold were grouped under “Other.” Asterisks indicate significant differences compared to the control (*p* < 0.05; DESeq2).

The relative abundance of microbial phyla, genera and species is shown in Figures 3–5. Metabarcoding analyses revealed differences in rhizosphere community composition and relative abundance compared with the control soil for each genotype, for both bacterial and fungal communities. In the figures, taxa that differ significantly between the rhizosphere and the control soil are indicated by an asterisk.

At the bacterial phylum level, *Pseudomonadota* (formerly *Proteobacteria*) (31.73–45.48%) and *Actinomycetota* (formerly *Actinobacteria*) (15.49–28.10%) were the most abundant taxa across all samples, while *Ascomycota* dominated the fungal communities (85.35–92.82%). The major bacterial and fungal phyla were consistently detected across all samples, indicating no major qualitative differences in phylum presence or absence. However, quantitative shifts in relative abundance were detected, particularly at the early developmental stage (T1). These changes were more pronounced for bacterial communities, with Far01 and Far10 showing significant variations in several bacterial and fungal phyla compared with the control soil (Figures 3A,C). At the second developmental stage (T2), substantially fewer differences were observed relative to the control (Figures 3B,D).

Genus-level analyses were subsequently used to obtain finer ecological and functional resolution of rhizosphere community assembly patterns. Several bacterial genera showed significantly increased relative abundance in rhizosphere samples (Figure 4), including taxa enriched across all three genotypes that may represent a common rhizosphere signature, such as *Sphingomonas* (3.64% for Far01, 3.67% for Far05, and 1% for Far10), *Massilia* (2.17, 3.90, and 2.06%, respectively) and members of *Oxalobacteraceae* (4.12, 6.91, and 3.17%, respectively), whereas others displayed genotype-specific enrichment patterns, including *Nocardioides* (7.11% for Far01 and 6.21% for Far10), *Marmoricola* (5.31% for Far10), *Geobacter* (4.17% for Far01 and 4.94% for Far10), and *Haliangium* (5.41% for Far10). Conversely, several bacterial and fungal taxa, including RB41, *Mortierella*, and *Micavibrionales*, showed reduced relative abundance compared with bulk soil, suggesting selective depletion from the rhizosphere environment. Significant quantitative changes are detailed in Tables 2, 3 and Supplementary Files 4, 5, together with literature-based functional annotations.

**TABLE 2:** Bacterial genera significantly enriched or depleted in the rhizosphere: common and genotype-specific signatures.

Bacterial genera	T1	T2	Associated plant genotype or bulk soil	Gram	Key references
*Altererythrobacter*	✓	–	Far01, Far05, Far10	–	(Kwon et al., 2007)
*Massilia*	✓	–	Far01, Far05, Far10	–	(Ofek et al., 2012)
*Oxalobacteraceae*	✓	✓	Far01, Far05, Far10	–	(Willems et al., 1991)
*Nocardioides*	✓	–	Far01, Far10	**+**	(Yoon et al., 1997)
*Sphingomonadaceae*	✓	–	Far01, Far10	–	(White et al., 1996; Fredrickson et al., 1995)
*Marmoricola*	✓	–	Far10	**+**	(Urzì et al., 2000)
OPS_17	–	✓	Far01, Far05	–	(Kielak et al., 2016)
*Cd. Yanofskybacteria*	–	✓	Far01, Far10	–	(Brown et al., 2015; Castelle and Banfield, 2018)
*Sphingomonas*	–	✓	Far01, Far05	–	(Takeuchi et al., 2001)
*Geobacter*	–	✓	Far01, Far10	–	(Lovley et al., 2004; Lovley, 1993)
*Flavobacterium*	–	✓	Far05, Far10	–	(McBride, 2014)
*Acidobacteriales*	–	✓	Far01	–	(Kielak et al., 2016)
*Actinocorallia*	–	✓	Far01	**+**	(Stackebrandt et al., 1997)
*Aquicella*	–	✓	Far01	–	(Saini and Gupta, 2021; Santos et al., 2003)
*Comamonadaceae*	–	✓	Far05	–	(Willems et al., 1991)
*Micrococcaceae*	–	✓	Far05	**+**	(Stackebrandt et al., 1997)
*Pseudarthrobacter*	–	✓	Far05	**+**	(Busse, 2016)
*Saccharimonadales*	–	✓	Far05	–	(Brown et al., 2015)
*Chryseolinea*	–	✓	Far10	–	(Yoon et al., 2011)
*Desulfuromonas*	–	✓	Far10	–	(Roden and Lovley, 1993)
*Haliangium*	–	✓	Far10	–	(Reichenbach, 2001; Phillips et al., 2022)
*Microscillaceae*	–	✓	Far10	–	(McBride, 2014)
*Ohtaekwangia*	–	✓	Far10	–	(Yoon et al., 2011)
*Saprospiraceae*	–	✓	Far10	–	(McBride, 2014)
A4b clade	–	✓	Far10	–	(Kielak et al., 2016)
RB41—Acidobacteriota	✓	–	Bulk soil Far10	–	(Kielak et al., 2016)
Subgroup 5—Acidobacteriota	–	✓	Bulk soil Far10	–	(Kielak et al., 2016)
*Micavibrionales*	–	✓	Bulk soil Far10	–	(Wang et al., 2011)

**TABLE 3:** Fungal genera significantly enriched or depleted in the rhizosphere: common and genotype-specific signatures.

Fungal genera	T1	T2	Associated plant genotype or bulk soil	Trophic strategy	Decomposition capacity	Key references
*Alternaria*	✓	✓	Far01, Far05, Far10	Pathotroph/saprotroph	Cellulose; simple plant polymers	(Thomma, 2003)
*Cladosporium*	✓	✓	Far01, Far05, Far10	Saprotroph/endophyte	Cellulose; leaf litter polymers	(Bensch et al., 2012)
*Dactylonectria*	✓	✓	Far01, Far05, Far10	Root pathogen	Root tissues; cellulose	(Lombard et al., 2015)
*Bionectriaceae*	–	✓	Far01	Saprotroph/pathotroph	Cellulose; plant debris	(Rossman et al., 1999)
*Helotiales*	–	✓	Far01	Endophyte/saprotroph	Root-derived carbon; simple polymers	(Hosoya, 2021)
*Spizellomycetales*	–	✓	Far01	Saprotroph	Simple carbohydrates	(Powell, 2017)
*Trichoderma*	–	✓	Far01	Mycoparasite	Cellulose; hemicellulose; chitin	(Harman et al., 2004)
*Lecanicillium*	–	✓	Far05	Entomopathogen	Chitin; insect cuticle; plant debris	(Zare and Gams, 2001)
*Neonectria*	–	✓	Far05	Pathotroph	Wood; lignocellulose	(Chaverri et al., 2011)
*Falciphora*	–	✓	Far10	Endophyte	Root-derived carbon	(Oberwinkler et al., 2013)
*Mortierella*	✓	–	Bulk soil Far10	Saprotroph	Lipids; simple plant polymers	(Ozimek and Hanaka, 2021)

After sequencing and OTU assignment, species-level annotation was achieved for 22.6% of bacterial OTUs at T1 and 3% at T2. For fungi, 49.7 and 46% of OTUs were identified at the species level at T1 and T2, respectively. Although species-level resolution remained limited, particularly for bacteria at T2, species-level ecological interpretations should be considered indicative. The relative abundances of the identified species are presented in Figure 5, highlighting both shared and genotype-dependent recruitment patterns. For example, species such as *Sphingomonas phyllosphaerae*, *Pseudochrobactrum asaccharolyticum*, and *Sporosarcina ureae* were enriched in rhizosphere samples, whereas other taxa, including *Streptomyces atrovirens*, *Streptomyces canus*, and *Exophiala radicis*, showed lower relative abundance compared with bulk soil. Significant quantitative changes are detailed in Tables 4, 5 and Supplementary Files 4, 5, together with literature-based functional annotations.

**TABLE 4:** Bacterial species significantly enriched or depleted in the rhizosphere: common and genotype-specific signatures.

Bacterial species	T1	T2	Associated plant genotype or bulk soil	Gram	Key references
*Pseudochrobactrum asaccharolyticum*	✓	–	Far01, Far05, Far10	–	(Kämpfer et al., 2006)
*Sphingomonas phyllosphaerae*	✓	–	Far01, Far05, Far10	–	(Takeuchi et al., 2001)
*Sporosarcina ureae*	✓	–	Far01, Far05, Far10	**+**	(Yoon et al., 2001)
*Streptomyces prasinus*	✓	–	Far01, Far05, Far10	**+**	(Hopwood, 2007)
*Symbiobacterium thermophilum*	✓	–	Far01, Far05, Far10	**+**	(Ueda et al., 2004)
*Thermobispora bispora*	✓	–	Far01, Far05, Far10	**+**	(Wang et al., 1997)
*Thermostaphylospora chromogena*	✓	–	Far01, Far05, Far10	**+**	BacDive
*Tumebacillus soli*	✓	–	Far01, Far05, Far10	**+**	(Kim and Kim, 2016)
*Streptomyces canus*	–	✓	Far01, Far05, Far10	**+**	(Bérdy, 2005)
*Streptomyces lincolnensis*	–	✓	Far01, Far05, Far10	**+**	(Bérdy, 2005)
*Oryzihumus leptocrescens*	–	✓	Far01, Far05, Far10	**+**	(Kageyama et al., 2005)
*Pseudarthrobacter oxydans*	–	✓	Far01, Far05, Far10	**+**	(Busse, 2016)
*Pseudarthrobacter siccitolerans*	–	✓	Far01, Far05, Far10	**+**	(Busse, 2016)
*Rhizorhabdus dicambivorans*	–	✓	Far01, Far05, Far10	–	(Yao et al., 2016)
*Sorangium cellulosum*	✓	–	Far01, Far05	–	(Reichenbach, 2001)
*Nitratireductor aestuarii*	✓	–	Far01, Far10	–	(Ou et al., 2017)
*Serratia plymuthica*	✓	–	Far01, Far10	–	(Proença et al., 2019)
*Thermoclostridium stercorarium* subsp. *leptospartum*	✓	–	Far01	**+**	(Poehlein et al., 2013)
*Thermoclostridium stercorarium* subsp. *thermolacticum*	✓	–	Far01	**+**	(Madden, 1983)
*Reyranella massiliensis*	–	✓	Far01	–	(Pagnier et al., 2011)
*Variovorax paradoxus*	–	✓	Far05	–	(Sun et al., 2018; Ma et al., 2023)
*Streptomyces caeruleatus*	–	✓	Far05	**+**	(Hopwood, 2007)
*Streptomyces ciscaucasicus*	–	✓	Far05	**+**	(Labeda et al., 2017)
*Bacillus cereus*	✓	–	Bulk soil Far01, Far05, Far10	**+**	(Kulkova et al., 2023)
*Bacillus drentensis*	✓	–	Bulk soil Far01, Far05, Far10	**+**	(Hernández-Pacheco et al., 2021)
*Bacillus frigoritolerans*	✓	✓	Bulk soil Far01, Far05, Far10	**+**	(Świątczak et al., 2024; Marik et al., 2024)
*Bacillus pseudomycoides*	✓	–	Bulk soil Far01, Far05, Far10	**+**	(Nakamura, 1998)
*Bacillus samanii*	✓	–	Bulk soil Far01, Far05, Far10	**+**	(Saman et al., 2010)
*Bacillus simplex*	✓	✓	Bulk soil Far01, Far05, Far10	**+**	(Karimi et al., 2024; Uroz et al., 2009)
*Clostridium vincentii*	✓	✓	Bulk soil Far01, Far05, Far10	**+**	(Mountfort et al., 1997)
*Ensifer adhaerens*	✓	–	Bulk soil Far01, Far05, Far10	–	(Zhou et al., 2013)
*Microlunatus panaciterrae*	✓	✓	Bulk soil Far01, Far05, Far10	**+**	(An et al., 2008)
*Oryzihumus leptocrescens*	✓	–	Bulk soil Far01, Far05, Far10	**+**	(Kageyama et al., 2005)
*Pseudarthrobacter oxydans*	✓	✓	Bulk soil Far01, Far05, Far10	**+**	(Busse, 2016)
*Pseudarthrobacter siccitolerans*	✓	✓	Bulk soil Far01, Far05, Far10	**+**	(Busse, 2016)
*Reyranella massiliensis*	✓	–	Bulk soil Far01, Far05, Far10	–	(Pagnier et al., 2011)
*Streptomyces atrovirens*	✓	–	Bulk soil Far01, Far05, Far10	**+**	(Hopwood, 2007)
*Streptomyces canus*	✓	–	Bulk soil Far01, Far05, Far10	**+**	(Bérdy, 2005)
*Streptomyces turgidiscabies*	✓	✓	Bulk soil Far01, Far05, Far10	**+**	(Loria et al., 2006)
*Variovorax paradoxus*	✓	–	Bulk soil Far01, Far05, Far10	–	(Sun et al., 2018; Ma et al., 2023)

**TABLE 5:** Fungal species significantly enriched or depleted in the rhizosphere: common and genotype-specific signatures.

Fungal species	T1	T2	Associated plant genotype or bulk soil	Trophic strategy	Decomposition capacity	Key references
*Pseudeurotium bakeri*	✓	✓	Far01, Far05	Saprotroph	Keratin; organic matter	(Adhikari et al., 2016)
*Pseudeurotium hygrophilum*	✓	✓	Far01, Far05	Saprotroph	Organic matter turnover	(Ogaki et al., 2020)
*Gibellulopsis nigrescens*	–	✓	Far01, Far10	Saprotroph/ weak pathotroph	Cellulose degradation	(Lombard et al., 2015)
*Lecanicillium subprimulinum*	–	✓	Far01	Entomopathogen/ mycoparasite	Chitin; insect cuticle; plant debris	(Zare and Gams, 2001)
*Exophiala radicis*	✓	–	Bulk soil Far01, Far05, Far10	Endophyte/ opportunist	Root-derived carbon; simple plant polymers	(Jumpponen and Trappe, 1998; de Hoog et al., 2011)
*Exophiala salmonis*	✓	–	Bulk soil Far01, Far05, Far10	Saprotroph/ opportunist	Simple organic substrates	(de Hoog et al., 2011)
*Gibellulopsis nigrescens*	✓	–	Bulk soil Far01, Far05, Far10	Saprotroph/ weak pathogen	Root tissues; cellulose	(Zare et al., 2007)
*Mortierella zonata*	✓	–	Bulk soil Far01, Far05, Far10	Saprotroph	Strong decomposer of plant polymers	(Wagner et al., 2013)
*Scytalidium lignicola*	✓	✓	Bulk soil Far01, Far05, Far10	Lignicolous saprotroph	Lignocellulose decomposition	(Fukasawa et al., 2011)
*Solicoccozyma aeria*	–	✓	Bulk soil Far01, Far05, Far10	Yeast-like saprotroph	Simple sugars; soluble carbon	(Liu et al., 2015)

### 3.4 RAST-based functional profiling of bacterial species associated with chicory genotypes

**FIGURE 6:**
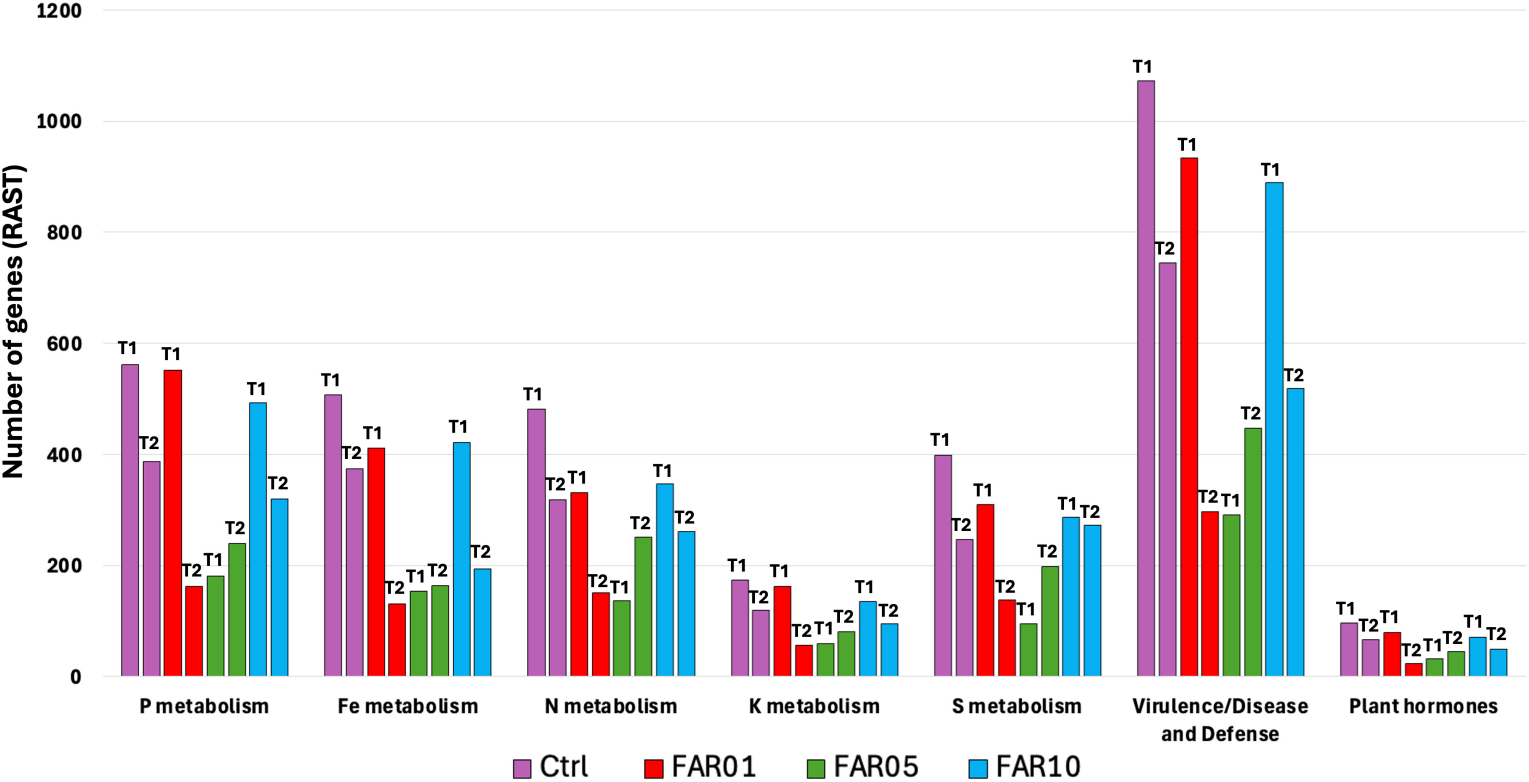
Number of bacterial genes involved in P, Fe, N, K, and S metabolism, virulence/defense, and plant hormone biosynthesis detected in rhizosphere and bulk soil (Ctrl) for the three genotypes (Far01, Far05, and Far10). Gene counts were obtained using RAST (https://rast.nmpdr.org/) for both developmental stages (T1 and T2).

The RAST server was used to perform putative functional characterization of representative genomes associated with the annotated bacterial species. To estimate putative beneficial functions, we quantified genes involved in phosphorus (P), iron (Fe), nitrogen (N), potassium (K), and sulfur (S) metabolism, as well as genes associated with virulence/defense mechanisms and plant hormone biosynthesis (Figure 6). For the bacterial species analyzed, we observed distinct and genotype-specific functional gene profiles. Although this analysis considers only a subset of the rhizosphere microbiota, and was based on literature-derived genome annotations, the results suggest potential genotype-dependent differences in bacterial recruitment patterns. At the early developmental stage (T1), the genotype Far10 showed putative functional profiles more similar to those of the bulk soil, a pattern that persisted at T2. In contrast, Far01 displayed, at T1, putative functional gene abundances comparable to those of the bulk soil, whereas at T2, bacterial recruitment appeared more restrictive. Conversely, Far05 exhibited an opposite trend relative to the other genotypes and the bulk soil, characterized by a more constrained recruitment at T1 and, overall, a markedly reduced abundance of functional genes across the functions investigated. Since strain-level genomic variation was not taken into account and gene abundance was not normalized across genomes, these analyses should be considered comparative and indicative rather than quantitative functional measurements.

### 3.5 NMR metabolic profiles of the three chicory genotypes

**FIGURE 7:**
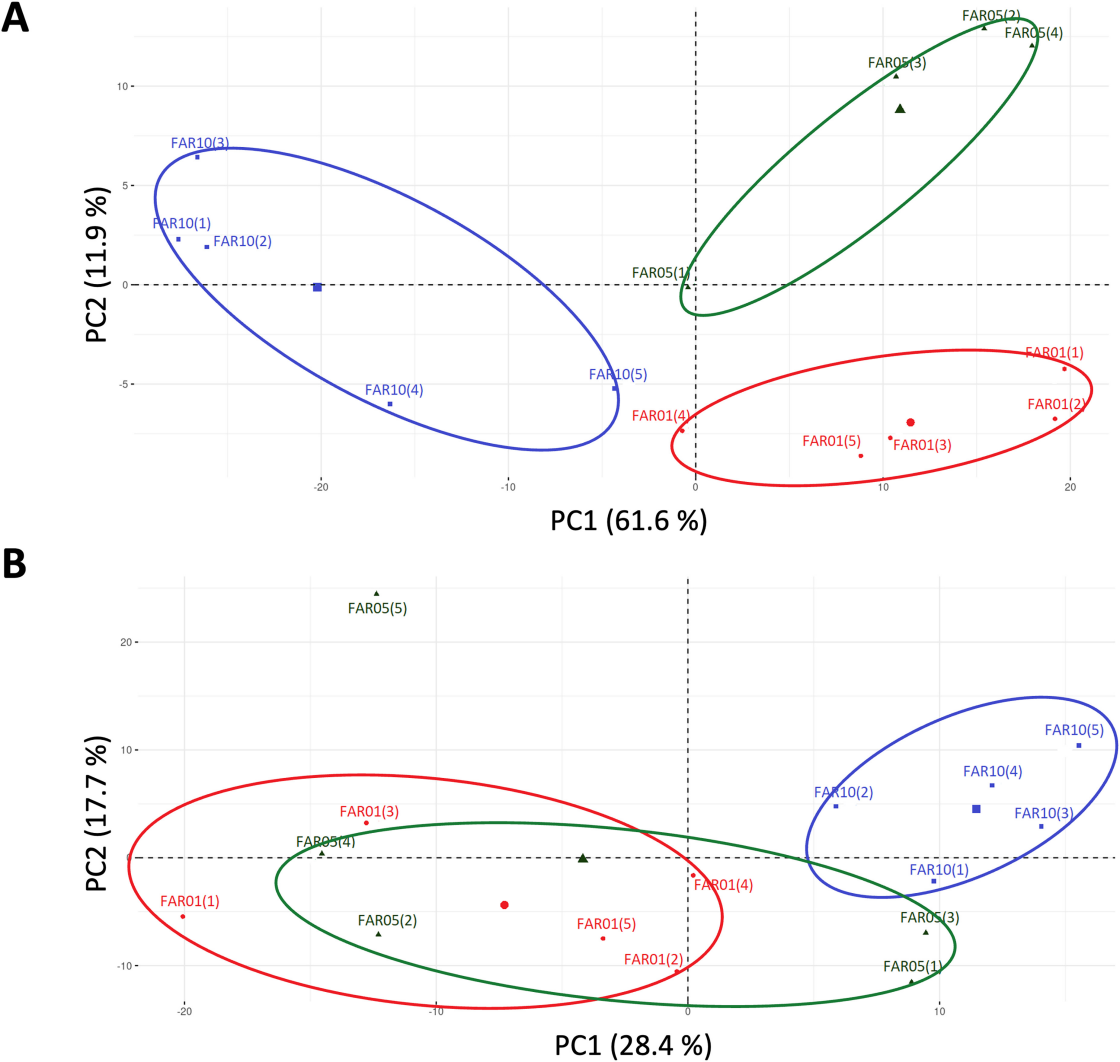
PCA score plots showing the distribution of samples based on metabolite profiles. PCA performed using NMR data illustrates the distribution of samples at T1 **(A)** and T2 **(B)**. Samples are colored according to genotype: Far01 (red), Far05 (green), and Far10 (blue). Ellipses are shown for visual guidance.

Detection and relative quantification of total metabolites were achieved using ^1^H NMR analysis. The acquired spectra resulted in a data matrix comprising 324 variables, which was subjected to principal component analysis (PCA) for samples collected at developmental stages T1 and T2 (Figure 7).

For T1 samples, the first two principal components explained 74% of the total variance, with PC1 accounting for 62% (Figure 7A). Intra-genotypic variability was limited across all genotypes, particularly for Far10, but also for Far05 and Far01, whereas inter-genotypic differences were more pronounced, allowing a clear discrimination among the three genotypes based on their total metabolite profiles.

For T2 samples, the first two principal components explained 46% of the total variance (Figure 7B). Far10 exhibited relative metabolic stability, as replicates clustered tightly and were clearly separated from the other samples. In contrast, Far01 and Far05 showed increased intra-genotypic variability, preventing a clear discrimination between these two genotypes based on total metabolite profiles. This pattern suggests that genotype-specific metabolic differences became less pronounced during root maturation.

Primary metabolites were identified by comparing the ^1^H NMR spectra and 2D NMR experiments (J-resolved and HSQC) of the QC sample with spectra acquired from reference standards available in an in-house library. Comparison of the J-resolved spectrum of the QC sample with that of sucrose revealed six characteristic resonances at 3.43, 3.50, 3.65, 4.03, 4.16, and 5.40 ppm, confirming the presence of sucrose in chicory roots. Using the same approach, additional metabolites, including glucose, glutamine, and malic acid, were identified. In addition, a broad carbohydrate signal pattern characteristic of inulin was observed in the spectra. Among these metabolites, all except the inulin-associated signal and malic acid were discriminant among the three chicory genotypes. Their relative levels were therefore compared by integrating well-resolved signals in the ^1^H NMR spectra. The signals at 5.40 ppm (d, 1H, ^3^J_H–H = 3.8 Hz) for sucrose, 5.18 ppm (d, 1H, ^3^J_H–H = 3.7 Hz) for glucose, and 2.17–2.08 ppm (m, 2H) for glutamine were selected for integration. At T1, significant genotype-dependent differences were observed for all three metabolites. Sucrose levels were significantly higher in Far01 and Far05 than in Far10, whereas α-glucose differed significantly between Far01 and Far10. Glutamine levels were significantly higher in Far05 than in both Far01 and Far10. In contrast, at T2, only sucrose showed significant differences among genotypes, whereas α-glucose and glutamine no longer differed significantly.

Because metabolomic analyses required destructive sampling of the root system, the same individual plants could not be analyzed at both developmental stages, and different individuals were therefore sampled at T1 and T2. In addition, T1 and T2 corresponded to distinct root developmental states (young fine roots versus mature tuberized roots). Consequently, part of the observed metabolic variation may reflect differences in root tissue physiology, storage capacity, and biochemical composition in addition to developmental and temporal effects.

### 3.6 Specialized metabolite profiles of the three chicory genotypes

While NMR analysis allowed the identification and relative quantification of major primary metabolites, its lower sensitivity for minor compounds led us to further investigate specialized metabolites using UHPLC–HRMS, which provides higher sensitivity and enhanced detection of secondary metabolites. UHPLC–HRMS analysis generated a data matrix comprising 148 variables, which was used to perform a principal component analysis (PCA) aimed at visualizing intra- and inter-genotypic variability in specialized metabolite profiles.

**FIGURE 8:**
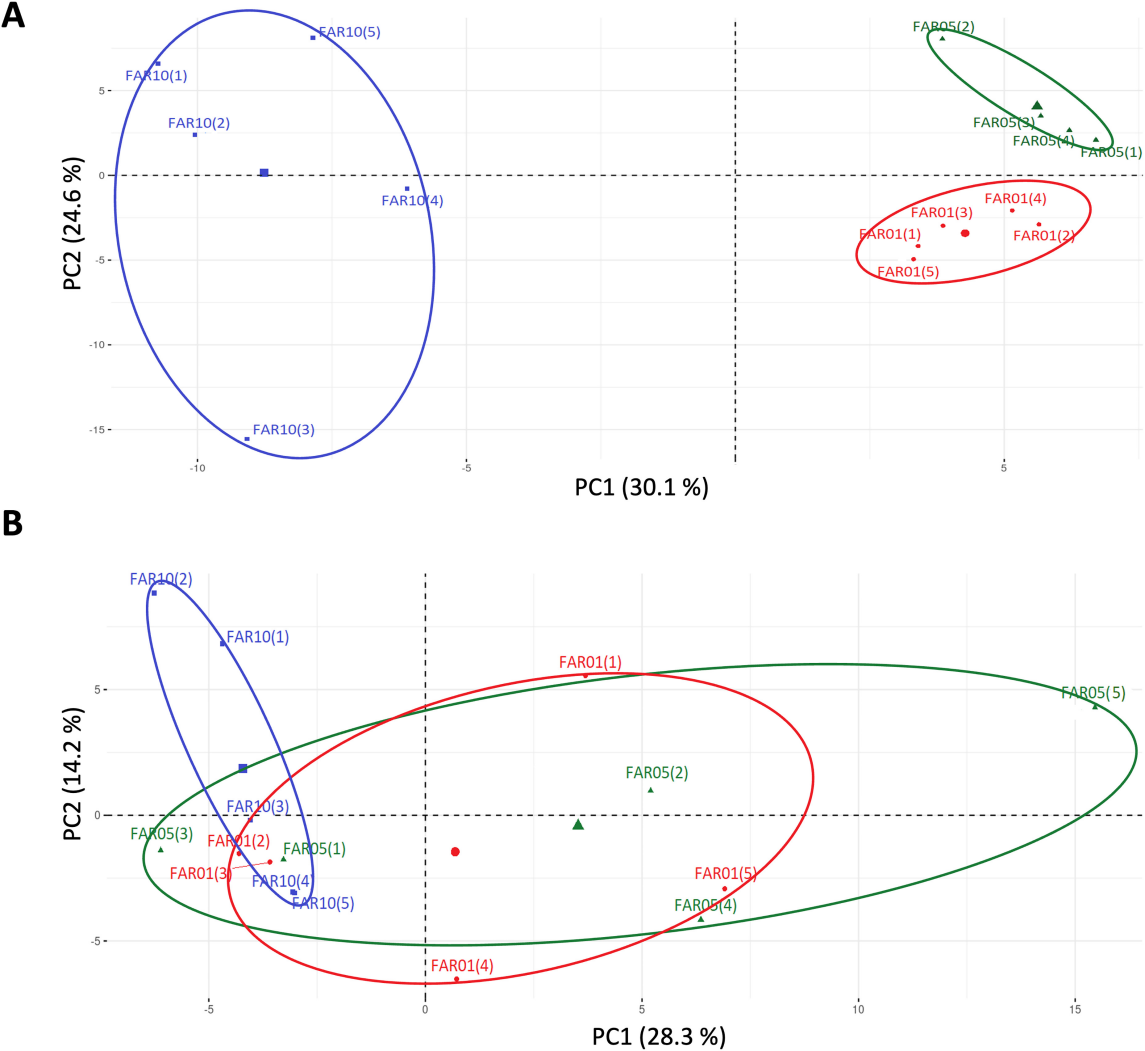
PCA score plots showing the distribution of samples based on specialized metabolite profiles. PCA performed using UHPLC–HRMS data illustrates the distribution of samples at T1 **(A)** and T2 **(B)**. Samples are colored according to genotype: Far01 (red), Far05 (green), and Far10 (blue). Ellipses are shown for visual guidance.

At the first developmental stage (T1), the first two principal components explained 55% of the total variance, with 30% accounted for by PC1 and 25% by PC2 (Figure 8A). Three distinct clusters corresponding to the Far01, Far05, and Far10 genotypes were observed, indicating low intra-genotypic variability and clear inter-genotypic differentiation at this developmental stage.

Metabolic profiles of tuberized roots at the second developmental stage (T2) are shown in Figure 8B. At this stage, the first two principal components accounted for 43% of the total variance. The first principal component, explaining 28% of the variance, revealed a highly variable distribution of specialized metabolites, particularly for Far01 and Far05. Overall, genotype-dependent differences were pronounced at T1 but became less distinct at the end of the developmental cycle (T2), suggesting a convergence of metabolic profiles across genotypes during root maturation.

Metabolite annotation was performed based on retention time, accurate mass, elemental composition, and MS^E^ fragmentation patterns. According to Metabolomics Standards Initiative (MSI) criteria, these annotations correspond to putatively annotated compounds (MSI level 2), as metabolite identities were not confirmed using authentic reference standards. The positions of annotated variables were then projected onto the PCA loading plots (Supplementary File 6), allowing the identification of 11 putative annotated variables potentially contributing to the discrimination among the three chicory genotypes. These variables correspond to features showing the highest contributions to the principal components and therefore likely represent metabolites involved in the metabolic differences observed between genotypes. Annotation of these markers (Table 6) enabled the identification of 11 metabolite features associated with genotypic differences among Far01, Far05, and Far10 at T1 and T2. All annotated markers were tentatively assigned to the sesquiterpene lactone family (STL), including guaianolides (including lactucin and lactucopicrin derivatives), germacranolides (parthenolide), and a putatively annotated eudesmanolide tentatively assigned as α-santonin, some of which occurred as oxalate or sulfate conjugates.

**TABLE 6:** Metabolite features associated with genotypic differences among Far01, Far05, and Far10 across both developmental stages, identified by LC–HRMS marker annotation.

Variable	Retention time (min)	[M + H] ^+^	[M - H] ^–^	Fragments	Molecular formula	Metabolite	Error (ppm)
V133	1.428	249.1497		231.1387–203.1436–185.1330–157.1020	C_15_H_20_O_3_	Parthenolide	0.3
V144	1.610	247.1328		229.1228–201.1284–183.1178–155.0862	C_15_H_18_O_3_	α-santonin	0.3
V186	2.117		347.0765	275.0815–257.0815–213.0915	C_17_H_16_O_8_	Lactucin-15-oxalate	0.6
V377	3.074		275.0921	257.0815–213.0916	C_15_H_16_O_5_	Lactucin	0.7
V419	3.855		469.2080	287.1283–247.1337–203.1439–185.1333	C_23_H_34_O_10_	6β-acetoxy-guai-9(10)- en12,8β-olide-4β-O-β- glucopyranoside	1.3
V474	4.511		339.0538	259.0977–257.0815 – 213.0915	C_15_H_16_O_7_S	15-Deoxylactucin-8-sulfate	0.0
V667	5.225		431.1105	409.1287–257.0815 –213.0915	C_25_H_20_O_7_	Lactucopicrin derivative	6.0
V861	5.430		331.0823	259.0977–215.1078	C_17_H_16_O_7_	8-Deoxylactucin-15-oxalate	1.5
V897	5.490		409.1298	257.0911–213.1013	C_23_H_22_O_7_	Lactucopicrin	1.5
V930	5.510		411.1457	259,1073–215.1161	C_23_H_24_O_7_	11β,13- Dihydrolactucopicrin	0.7
V1111	5.578		481.1151	409.1450–257.0911–213.1012	C_25_H_22_O_9_	Lactucopicrin-15-oxalate	3.5

**FIGURE 9:**
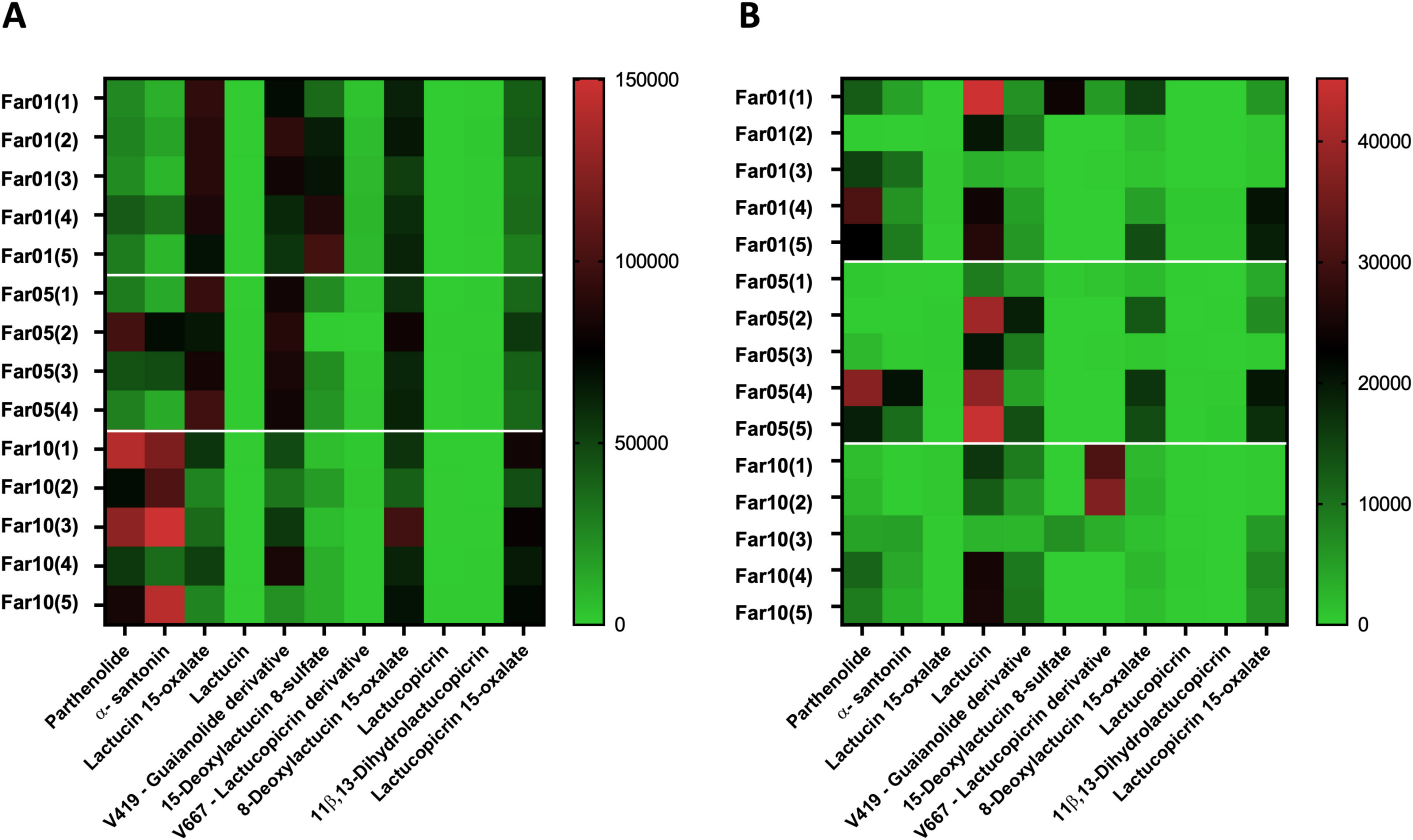
Heatmaps of metabolite markers contributing to variability in chicory roots based on specialized metabolite profiles. Each sample (1–5) from each genotype (Far01, Far05, and Far10) was analyzed at two harvest times, T1 **(A)** and T2 **(B)**. Red indicates high metabolite abundance, whereas green indicates low abundance, based on signal intensities.

Comparison of marker abundances across T1 and T2 samples enabled the identification of major genotypic differences in metabolite quantification, visualized using a heatmap (Figure 9). At T1, several metabolites, including parthenolide, α-santonin, lactucin 15-oxalate, guaianolide derivative, and lactucopicrin 15-oxalate, showed the strongest differences among the three genotypes. At T2, most of these differences were attenuated, leading to a more homogeneous metabolic profile across genotypes, except for lactucin, which exhibited the most pronounced differences. Lactucopicrin and 11β,13-dihydrolactucopicrin displayed the most consistent temporal trends across genotypes. Overall, lower signal intensities were observed at T2, with maximum signal values of approximately 40,000 compared with about 150,000 in T1 samples. However, these differences should not be interpreted as direct evidence of decreased metabolite concentrations, as developmental changes in root biomass and tissue composition may also have contributed to the observed patterns.

### 3.7 Stage-dependent microbiome–metabolome correlations

To investigate relationships between rhizosphere microbial communities and specialized metabolites in chicory roots, we combined multivariate and pairwise correlation approaches.

**FIGURE 10:**
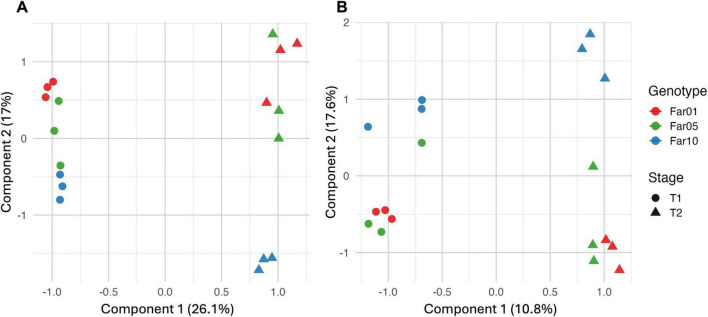
Correlation analysis between significant discriminant bacterial **(A)** and fungal **(B)** genera and STL metabolite markers. Regularized canonical correlation analysis (rCCA) was performed on paired samples to assess relationships between microbial community composition and specialized metabolite profiles. Points represent individual samples, colored by genotype (Far01, Far05, Far10) and shaped according to developmental stage (T1, T2). Axes indicate the percentage of variance explained.

Regularized canonical correlation analysis (rCCA) suggested multivariate co-variation between microbial genera and metabolite profiles (Figure 10). In both bacterial and fungal datasets, samples were primarily separated along the first canonical component (26.1% of the microbial variance in bacteria, 10.8% in fungi), according to developmental stage, indicating that shifts in metabolite composition and microbial community structureshowed coordinated variation over plant development. The second component (17 and 17.6%, respectively) appeared to reflect genotype-related variation, although this effect was less pronounced than that of stage. The metabolites potentially contributing to the canonical structure included lactucin, lactucopicrin, 11,13-dihydrolactucopicrin, α-santonin, and lactucin 15-oxalate. The metabolite subset included the annotated specialized metabolites showing the highest contributions to PCA discrimination and the most consistent variation across genotypes and developmental stages. Among microbial taxa, the main contributors were *Geobacter*, *Desulfuromonas*, *Micrococcaceae*, *Acidobacteriales*, and *Saprospiraceae* for bacteria, and *Trichoderma*, *Neonectria*, *Dactylonectria*, *Lecanicillium*, and *Helotiales* for fungi.

**FIGURE 11:**
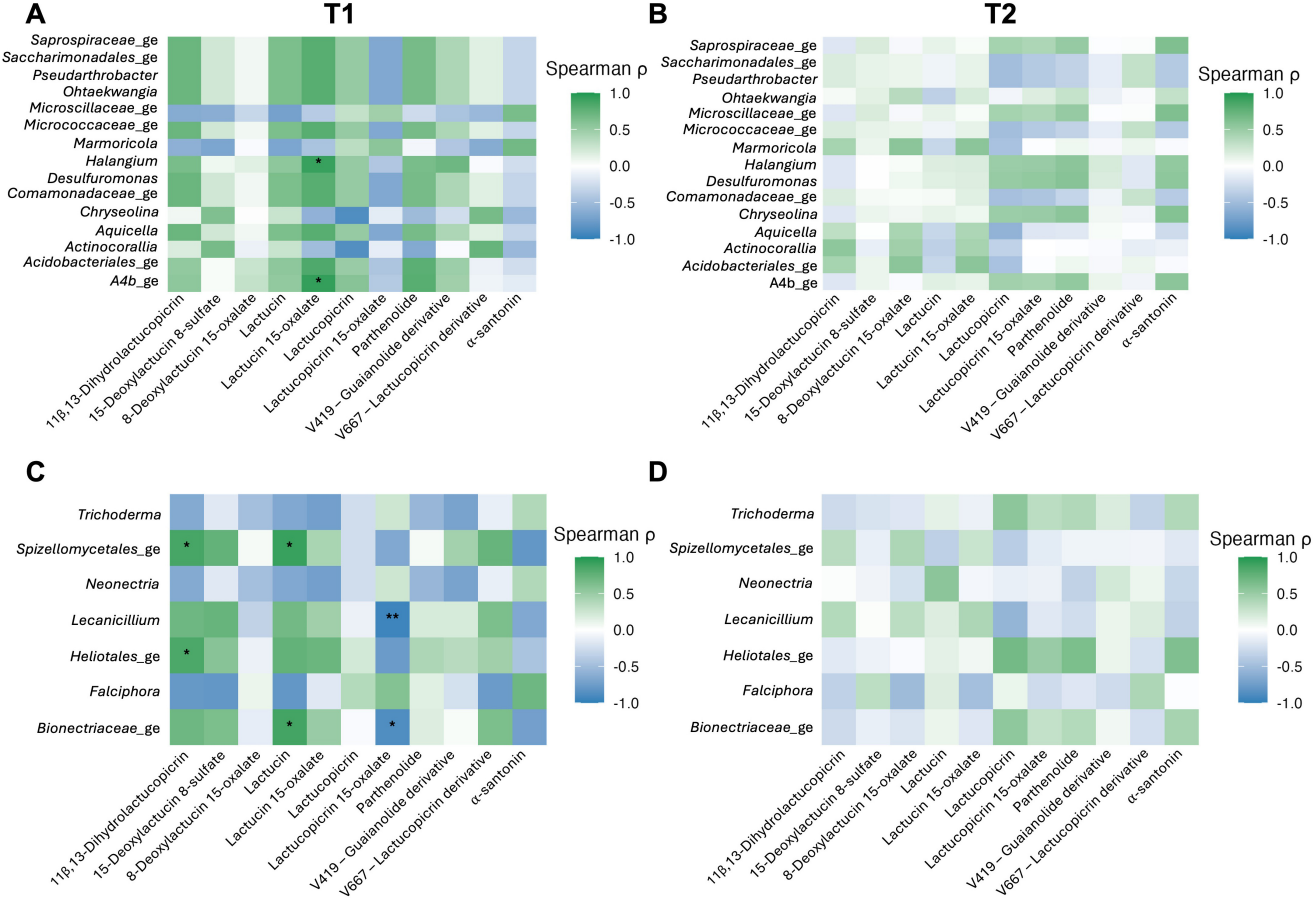
Heatmaps showing Spearman correlation coefficients (ρ) between bacterial **(A,B)** and fungal **(C,D)** genus abundances and specialized metabolites in chicory at two developmental stages (T1 and T2). Colors represent the strength and direction of the associations (blue—negative correlation, green—positive correlation, white—no correlation). *P*-values were adjusted for multiple testing using the Benjamini–Hochberg false discovery rate (FDR) procedure. Asterisks indicate statistically significant correlations after FDR correction (adjusted *p* < 0.05).

To further examine specific genus–metabolite associations, Spearman correlation analyses were performed separately for each developmental stage (Figure 11). Within the bacterial community, two significant correlations were identified at the early stage (T1) after FDR correction (adjusted *p* < 0.05): Ab4 (ρ = 0.93; *p* = 0.04) and *Haliangium* (ρ = 0.92; *p* = 0.04) were positively correlated with lactucin15-oxalate. No significant bacterial associations were detected at the later stage (T2).

In the fungal community, six significant correlations were observed at T1. *Spizellomycetales* (ρ = 0.92; *p* = 0.02) and *Bionectriaceae* (ρ = 0.88; *p* = 0.03) were positively correlated with lactucin, whereas *Lecanicillium* (ρ = -0.95; *p* = 0.007) and *Bionectriaceae* (ρ = -0.88, *p* = 0.03) were negatively correlated with lactucopicrin-15-oxalate. Additionally, *Spizellomycetales* (ρ = 0.87; *p* = 0.04) and *Helotiales* (ρ = 0.85; *p* = 0.05) were positively correlated with 11β,13-dihydrolactucopicrin. Effect sizes and 95% bootstrap confidence intervals for all significant pairs are reported in Supplementary File 7. As for bacteria, no significant correlations remained at T2 after FDR correction.

Overall, correlation strength and the number of significant associations were markedly higher at T1 than at T2. Statistical power was constrained by the modest samples sizes (*n* = 18 for rCCA, *n* = 9 per stage for Spearman). Consequently, the rCCA approach was ridge-regularized and should be considered exploratory, while the bootstrap confidence intervals associated with Spearman correlations remained relatively wide. Together with the rCCA results, these findings suggest that microbiome–metabolome relationships are predominantly stage-dependent and appeared more pronounced during early plant development. However, the limited number of significant pairwise associations remaining after FDR correction suggests that the observed microbiome–metabolome co-variation largely reflects shared structuring factors, notably developmental stage and genotype, rather than direct interactions between individual genera and specific metabolites. Although our data do not demonstrate a direct causal role of STLs in microbial recruitment, these observations support their potential contribution to rhizosphere filtering and suggest that STLs may contribute to shaping the chemical environment associated with microbial assembly.

## 4 Discussion

### 4.1 Specialization rather than increased taxonomic diversity drives community assembly

The rhizosphere is widely recognized as a hotspot of microbial activity due to the continuous release of root exudates, which strongly influence microbial abundance and community assembly. The reduced α-diversity observed in rhizosphere samples, particularly at the early developmental stage, suggests a strong selective filtering exerted by chicory roots. These effects were genotype-dependent and were more pronounced at T1 than at the later developmental stage (T2) (Figure 1). Numerous studies have shown that rhizosphere microbial communities differ markedly from those of bulk soil, although the effect of the rhizosphere on microbial α-diversity remains variable and depends on plant species, soil properties, and developmental stage. While some studies have reported higher microbial richness in the rhizosphere relative to bulk soil (Mendes et al., 2011; Shi et al., 2015), others have consistently observed reduced α-diversity indices, such as Shannon or Simpson, in rhizosphere communities (Haichar et al., 2008; Chaparro et al., 2014). This reduction is generally attributed to the selective enrichment of specific microbial taxa favored by root exudates, leading to increased community specialization and reduced evenness rather than a loss of functional potential. Accordingly, a lower α-diversity in the rhizosphere does not contradict the rhizosphere effect but instead reflects a deterministic filtering process through which plants actively shape a specialized microbial consortium adapted to the rhizosphere environment (Bulgarelli et al., 2013; Fitzpatrick et al., 2018).

Consistent with this framework, the systematically lower α-diversity observed in rhizosphere samples in the present study, particularly at T1, supports a model of plant-driven microbial selection in which chicory selectively recruits a specialized microbial consortium rather than promoting increased taxonomic diversity. These results further suggest that early developmental stages in chicory are characterized by stronger selective pressures on the rhizosphere microbiome than later stages of development.

### 4.2 Genotype- and developmental stage-dependent assembly of the rhizosphere microbiome

The observed community structuring supports the idea that both plant genotype and developmental stage act as major determinants of rhizosphere assembly (Figure 2 and Supplementary File 3). Metabarcoding analyses further showed genotype-dependent differences in rhizosphere bacterial and fungal community composition relative to the bulk soil control, with each genotype recruiting significantly different taxa at each developmental stage (Figures 3–5 and Supplementary Files 4, 5). These findings are consistent with numerous studies demonstrating that host plant genotype acts as a deterministic filter, selectively shaping microbial communities from the soil pool (Bulgarelli et al., 2013). Genotypic effects have been reported across multiple plant species, including *Arabidopsis thaliana*, maize, and wheat, where distinct genotypes recruit different subsets of bacteria and fungi to their rhizosphere (Schlaeppi et al., 2014; Tkacz et al., 2015; Hartman et al., 2017). These differences are often linked to variations in root exudate composition, which modulate the recruitment of microbial consortia.

The observed stage-dependent clustering further highlights the dynamic nature of rhizosphere microbial communities. Early developmental stages are frequently characterized by strong selective pressures, where root exudates selectively enrich specific fast-growing microbial taxa (Chaparro et al., 2014; Shi et al., 2015). Later developmental stages often show more stable communities with gradual shifts toward taxa adapted to nutrient cycling and plant defense, reflecting successional assembly processes (Shi et al., 2015; Mendes et al., 2011).

In our study, this is supported by root metabolite profiles, which were more discriminative among the three genotypes at the early developmental stage (Figures 7–9). Consistent with this coordinated structuring, multivariate and pairwise correlation analyses further revealed stage-dependent microbiome–metabolome associations (Figures 10, 11), indicating that shifts in microbial community composition and specialized metabolite profiles appear to be closely associated, particularly during early plant development. Overall, our findings reinforce the view that rhizosphere microbial communities are co-structured by plant genotype and developmental cues, in accordance with root exudate composition.

### 4.3 Genotype-modulated temporal bacterial rhizosphere filtering

Microbial recruitment by chicory rhizospheres throughout the plant life cycle was inferred based on genera and species identified as significantly enriched in the rhizosphere or, conversely, significantly depleted relative to bulk soil (Tables 2–5; Supplementary Files 4, 5). Genus-level interpretation provides ecological robustness, whereas species-level analysis, although limited by incomplete taxonomic resolution, allows greater mechanistic precision in inferring genotype-specific strategies among the three chicory genotypes. Overall, the observed microbial patterns were consistent with coherent ecological trends inferred from the literature. A shared recruitment signature was observed across all three genotypes, alongside genotype-specific particularities.

For bacterial taxa selectively filtered by the rhizosphere, the interpretation was based on the significant changes reported in Tables 2, 4 and on the comprehensive functional features described in Supplementary File 4.

#### 4.3.1 Excluded taxa: active ecological filtering

Taxa significantly more abundant in bulk soil than in the rhizosphere were operationally considered as depleted from the rhizosphere. At T1, the RB41 (an uncultured lineage affiliated with *Acidobacteriota*) showed lower relative abundance in the rhizosphere, while at T2, Subgroup 5 within *Acidobacteriota* and *Micavibrionales* were significantly depleted, particularly in the Far10 genotype. These taxa consistently clustered within oligotrophic bulk-soil specialists, aggressive Gram-positive competitors, and lignocellulolytic saprotrophs, suggesting that chicory does not merely enrich compatible microbes but actively limits the development of structurally degradative and highly competitive taxa.

At the species level, a common depletion pattern was observed across the three genotypes, especially at T1. Chicory preferentially repelled Gram-positive bacteria, aggressive secondary metabolite producers (e.g., *Streptomyces atrovirens*, *Streptomyces canus*), as well as many competitive taxa, and phytopathogens (e.g., *Streptomyces turgidiscabies*). This pattern suggests selection toward a more specialized and less antagonistic microbiome. Such selective pressure is likely mediated through root exudates that impose trophic constraints disadvantaging these taxa. This pattern was also reflected in rhizosphere functional analyses (Figure 6), where genes associated with diverse metabolic pathways, particularly virulence-related genes, were more abundant in bulk soil than in all tested rhizospheres.

#### 4.3.2 Early-stage recruitment: nutrient support and protection

At the early growth stage (T1), chicory rhizospheres were enriched with bacteria primarily functioning as controlled polymer degraders, aromatic compound metabolizers, nitrogen cycle modulators, and antifungal metabolite producers. A conserved recruitment signature was evident at both genus and species levels. *Altererythrobacter*, *Massilia*, and members of *Oxalobacteraceae* were selected for their capacity to solubilize phosphate and recycle nutrients. Far01 and Far10 showed particularly strong early recruitment of taxa such as *Nocardioides*, *Sphingomonadaceae*, and *Marmoricola*, also known for phosphorus solubilization and carbon recycling.

Eight species were identified within the shared early-stage signature, including *Sphingomonas phyllosphaerae*, known for aromatic compound metabolism, and *Pseudochrobactrum asaccharolyticum*, capable of organic matter transformation. *Sporosarcina ureae* contributed nitrogen transformation capacity, while *Thermobispora* and *Thermostaphylospora* are actinomycete genera involved in organic matter degradation and recycling. In addition, *Streptomyces prasinus*, a known antibiotic producer, was enriched. Together, these taxa indicate that early-stage recruitment fulfills both nutritional requirements and antipathogenic protection across genotypes.

Genotype-specific differences were also observed at the species level during early recruitment, although associated taxa retained similar functional roles. These included nitrogen supply (*Nitratireductor aestuarii*), carbon recycling through strong cellulolytic activity (*Sorangium cellulosum*, *Thermoclostridium stercorarium*), and protective functions (*Serratia plymuthica* with antifungal activity; *Sorangium cellulosum* producing antimicrobial secondary metabolites). Early recruitment was most pronounced in Far01, whereas Far05 displayed comparatively limited recruitment pattern.

#### 4.3.3 Late-stage recruitment: transition toward controlled saprotrophy

Whereas early-stage recruitment (T1) reflected active root growth and nutrient acquisition, late-stage recruitment (T2) corresponded to a transition toward saprotrophic community dynamics. Plant senescence led to accumulation of dead tissues, xenobiotic compounds, and increased abiotic and biotic stress. Accordingly, the shared late-stage signature included species such as *Pseudarthrobacter* spp., capable of oxidizing organic compounds; *Rhizorhabdus dicambivorans*, known for herbicide degradation; and antibiotic producers such as *Streptomyces canus* and *Streptomyces lincolnensis*.

Comparative analysis of rhizosphere bacterial communities at the late developmental stage further revealed genotype-associated differences in community composition. Far01 preferentially enriched taxa such as *Acidobacteriales*, *Actinocorallia*, and *Aquicella*, together with shared enrichment of *Geobacter* and *Sphingomonas*. These taxa are associated with oligotrophic environments, iron cycling, and aromatic compound degradation, suggesting that Far01 promotes a microbiome adapted to nutrient-limited conditions and finely regulated carbon turnover.

Far05 was characterized by higher relative abundance of *Comamonadaceae*, *Micrococcaceae*, *Pseudarthrobacter*, *Saccharimonadales*, and *Flavobacterium*. Many of these taxa metabolize complex organic substrates and thrive in carbon-enriched environments, suggesting that this genotype may favor communities adapted to enhanced microbial turnover during senescence.

Far10 displayed enrichment of taxa including *Chryseolinea*, *Desulfuromonas*, *Haliangium*, *Microscillaceae*, *Ohtaekwangia*, and *Saprospiraceae*, several of which are involved in sulfur cycling, polymer degradation, and microbial interactions such as predation. This pattern suggests a shift toward a more interaction-driven and decomposition-associated rhizosphere network. Partial overlap among genotypes (e.g., *Geobacter*, *Sphingomonas*, *Flavobacterium*) indicates the presence of a shared functional core microbiota modulated by host genotype.

Species-level analyses broadly mirrored genus-level patterns, providing complementary but more tentative support for genotype-associated community structuring. Although late-stage sampling likely integrates decomposition-driven dynamics, congruence across taxonomic resolutions strengthens evidence for genotype-dependent ecological filtering. Specifically, *Reyranella massiliensis* was enriched in Far01, consistent with persistence of oligotrophic and resource-efficient bacteria favored by genotype-specific residue chemistry. In contrast, Far05 selectively enriched *Variovorax paradoxus* and two *Streptomyces* species (*S. caeruleatus*, *S. ciscaucasicus*). *Variovorax paradoxus*, a metabolically versatile rhizosphere bacterium involved in organic compound turnover and plant signaling modulation, may benefit from increased soluble substrates during tissue deterioration. The enrichment of *Streptomyces* spp., known for complex polymer degradation and secondary metabolite production, suggests a shift toward decomposition-oriented and competitive microbial dynamics.

Overall, these results are consistent with a non-random bacterial filtering process in chicory rhizospheres, potentially associated with coherent ecological functions inferred from the literature. The observed recruitment and depletion patterns may reflect coordinated shifts related to nutrient acquisition, microbial compatibility, and carbon turnover across developmental stages, while remaining quantitatively modulated by genotype-specific traits.

### 4.4 Genotype-modulated temporal fungal rhizosphere filtering

For fungal taxa selectively filtered by the rhizosphere, the interpretation was based on the significant changes reported in Tables 3, 5 and on the comprehensive functional features described in Supplementary File 5. Functional interpretations of fungal taxa were inferred from taxonomic identity and previously published ecological descriptions and should therefore be considered putative rather than directly demonstrated within the present study.

#### 4.4.1 Fungal exclusion and chemical control

At the early growth stage (T1), a selective exclusion of fungal taxa was observed, particularly in Far10 at the genus level (*Mortierella*) and across all chicory genotypes at the species level. Notably, no major chicory-specific pathogens were excluded. Instead, the plant appeared to restrict robust and competitive saprotrophs, melanized stress-resistant fungi, species with high extracellular enzymatic potential, and opportunistic root colonizers.

Two *Exophiala* species, *Exophiala radicis* and *Exophiala salmonis*, were significantly excluded at T1 by all chicory genotypes. Both species are known as opportunistic root-colonizing endophytes. Their consistent early exclusion suggests the existence of a selective chemical environment in the rhizosphere, potentially associated with genotype-dependent specialized metabolite profiles, including STL-rich exudates, with known antifungal properties, and possibly reinforced by antifungal metabolites produced by concurrently recruited rhizosphere bacteria such as *Serratia plymuthica*, *Sorangium cellulosum*, and *Streptomyces prasinus*. Three additional fungal species, *Gibellulopsis nigrescens*, *Mortierella zonata*, and *Scytalidium lignicola*, were also excluded at T1. These are soil saprotrophs characterized by strong organic matter degradation capacities. Their exclusion suggests that chicory rhizosphere may create ecological conditions that limit early competition for root-derived carbon, prevent premature tissue degradation, andreduce excessive carbon mineralization, although the underlying mechanisms remain hypothetical.

At the late developmental stage (T2), chicory exhibited changes in its metabolite profile (Figures 8, 9), and fungal exclusion was less pronounced. Only two saprotrophic species, *Scytalidium lignicola* and *Solicoccozyma aeria*, remained significantly excluded. This shift coincided with changes in the functional profile of the associated bacterial microbiome, suggesting a coordinated modulation of bacterial–fungal interactions during senescence.

#### 4.4.2 Early-stage recruitment: functional compatibility

Concerning rhizosphere recruitment, across the three chicory genotypes, a conserved rhizosphere fungal signature emerged. All genotypes consistently harbored genera containing potential pathogens such as *Alternaria*, *Cladosporium*, and *Dactylonectria*. These taxa were not fully excluded but maintained at controlled levels, indicating that chicory does not establish a sterile rhizosphere but rather maintains a diverse microbial equilibrium.

At the species level, *Pseudeurotium bakeri* and *Pseudeurotium hygrophilum* were significantly associated with Far01 and Far05 at T1. These soil ascomycetes are moderate saprotrophs known for tolerance to cold and nutrient-poor environments. Their persistence at T2 suggests strong compatibility with the chicory rhizosphere, likely functioning as stable commensals capable of utilizing both exudates and senescent residues.

#### 4.4.3 Late-stage recruitment: relaxation and controlled trophic opening

The genotype-dependent modulation became more apparent during the late stage. Far01 showed recruitment of additional saprotrophic taxa (e.g., *Bionectriaceae*, *Helotiales*, *Spizellomycetales*, and *Trichoderma*). Far05 recruited *Lecanicillium* (saprotrophic) and *Neonectria* (pathotrophic), while Far10 favored the endophyte *Falciphora*.

The species-level recruitment at T2 showed that Far01 and Far10 displayed recruitment of additional taxa, including *Gibellulopsis nigrescens*, previously excluded at T1. Its later emergence can be explained by its strong saprotrophic capacity and competitive advantage on senescent tissues. Moreover, *Lecanicillium subprimulinum* was recruited by Far01 at T2. This species, known as an entomopathogen and occasional mycoparasite, may contribute to fungal regulation and biological control within the rhizosphere.

These fungal dynamics suggest that during early growth, chicory recruits non-aggressive saprotrophs with low pathogenic potential while competitive or highly degradative fungi were depleted from the rhizosphere community. During late development, the plant maintains compatible saprotrophs and permits the arrival of more active decomposers, while still exerting antifungal regulation. Although this overarching pattern is conserved across genotypes, its apparent intensity and trophic openness appear to be modulated in a genotype-dependent manner. Altogether, these findings are consistent with a temporally structured fungal filtering pattern associated with bacterial community shifts and dynamic changes in root metabolite composition and putative exudation patterns, which may contribute to controlled carbon turnover and overall rhizosphere functional stability.

### 4.5 Conceptual model of temporally structured chemical-trophic filtering in chicory rhizosphere

The physicochemical properties of the soil define an abiotic framework within which rhizosphere microbial communities assemble. The soil supporting chicory growth was characterized by near-neutral pH (pH*water* = 7.04), low total nitrogen content (0.15%), moderate total organic carbon (2.22%), and a mineral composition dominated by aluminosilicates, with relatively low CaO content (1.15%) (Table 1). This geochemical signature indicates a predominantly feldspar- and clay-bearing mineral substrate with limited carbonate buffering capacity and moderate contributions from clay-associated fractions. Such conditions are typically associated with moderate nutrient availability and may favor microorganisms adapted to relatively constrained resource environments. Within this context, plant-associated processes are expected to contribute to additional structuring of microbial communities.

Across genotypes and developmental stages, the observed shifts in microbial community composition and relative abundance indicate non-random assembly patterns in the chicory rhizosphere. During active growth, the rhizosphere was enriched in microbial groups reported in the literature to contribute to nutrient mobilization and microbial interactions, including nitrogen cycle-associated taxa (e.g., *Nitratireductor*, *Sporosarcina*), aromatic and polymer degraders (*Sphingomonas*, *Variovorax*, *Sorangium*), taxa with reported antagonistic potential (*Serratia*, *Streptomyces prasinus*), and moderate saprophytic fungi such as *Pseudeurotium* spp. (Tables 2–5 and Supplementary Files 4, 5). Although these functional roles were not directly measured, their consistent enrichment suggests that the rhizosphere may favor a functionally constrained consortium associated with nutrient acquisition and regulated microbial interactions.

At later developmental stages, the accumulation of senescent tissues likely introduces new substrates into the rhizosphere. The enrichment of saprotrophic Actinobacteria such as *Streptomyces* spp., aromatic degraders such as *Pseudarthrobacter*, and other decomposers, together with the partial reappearance of previously less abundant taxa such as *Gibellulopsis* and the recruitment of fungal regulators including *Lecanicillium*, indicates a controlled opening of trophic niches (Tables 2–5 and Supplementary Files 4, 5). Such patterns are consistent with a relaxation of early-stage selective pressure, and the emergence of communities adapted to an increased availability of plant-derived substrates associated with root maturation and senescence. This shift is consistent with principles of microbial succession and community reorganization during resource changes (DeAngelis et al., 2009; Shade et al., 2013).

Genotype-dependent differences were observed throughout this process, affecting both microbial composition and metabolite profiles. Although the overall patterns of community assembly were broadly conserved, genotypes differed in the intensity and timing of these changes.

Taken together, the results are consistent with a conceptual model in which chicory rhizosphere assembly follows a temporally structured and genotype-modulated filtering process associated with both soil constraints and plant metabolic variation, in line with conceptual models of chemical-trophic filtering proposed for other plant species (Lattanzio et al., 2006; Bais et al., 2006). Our observations are consistent with the hypothesis that chicory rhizosphere assembly may be influenced by multiple interacting factors, including physicochemical soil constraints, trophic inputs associated with primary metabolites, STL-associated selective pressures, microbial interactions, and stage-dependent trophic reorganization. These patterns may also interact with the pre-existing soil microbial reservoir and potential soil legacy effects associated with previous cropping history. Within this conceptual framework, chicory-associated microbiome dynamics could contribute to a potentially disease-suppressive rhizosphere state. If confirmed experimentally, such suppressiveness would likely result from complex interactions among carbon availability, nutrient dynamics, and microbial competition rather than from simple antagonistic mechanisms alone. Genotype-dependent differences may further modulate the degree of rhizosphere openness while maintaining broadly similar ecological trends across genotypes.

From an agronomic perspective, these findings suggest that rhizosphere recruitment patterns could represent candidate traits of interest for future chicory breeding programs. Genotype-dependent differences in the recruitment of microbial consortia potentially associated with nutrient mobilization, microbial interactions, or decomposition processes may contribute to plant adaptation and resilience, although these putative functions remain to be experimentally validated. More broadly, integrating microbiome-associated traits into breeding strategies could provide new perspectives for developing chicory cultivars with enhanced microbiome-associated functions and potentially improved agroecosystem sustainability. Chicory could also represent a promising candidate service plant for intercropping or cover-cropping systems, where its influence on rhizosphere microbial communities might contribute to soil functioning, nutrient cycling, and microbiome dynamics associated with subsequent crops. However, these potential agronomic applications will require validation under controlled and field conditions.

Beyond its agronomic importance, chicory is widely used as a functional food, coffee substitute, and medicinal plant due to its high content of bioactive compounds, including inulin, phenolic compounds, and STLs (Puhlmann and de Vos, 2021). The observed associations between root metabolite profiles and rhizosphere microbial communities therefore suggest that plant phytochemistry may contribute not only to rhizosphere ecology but also potentially to traits associated with plant quality. Nevertheless, the mechanistic relationships between chicory phytochemistry, metabolite exudation, and microbiome assembly remain unresolved and will require further studies combining rhizosphere microbiology, metabolomics, exudate characterization, and functional bioassays.

## 5 Conclusion

This study shows that rhizosphere microbial communities associated with chicory differ consistently from bulk soil and vary according to plant genotype and developmental stage. The reduced α-diversity observed at early developmental stages is consistent with selective enrichment of specific microbial taxa, suggesting non-random assembly patterns in the rhizosphere. The integration of metabarcoding and metabolomic data revealed that variation in microbial community composition was associated with variation in root metabolite profiles, including both primary metabolites and specialized compounds such as STLs. These associations were particularly pronounced at early developmental stages, when both microbial and metabolic differentiation among genotypes were strongest.

Across developmental stages, the rhizosphere microbiome shifted from communities enriched in taxa reported to be associated with nutrient transformation and microbial interactions toward communities increasingly characterized by saprotrophic and organic matter-degrading taxa. This transition is consistent with changes in plant developmental status and resource availability and may be interpreted within a conceptual model of a temporally structured chemical-trophic filtering process.

Genotype-dependent differences in both microbial and metabolite profiles further suggest that plant genetic background may influence rhizosphere assembly.

Overall, the observed associations are consistent with a temporally structured and genotype-modulated rhizosphere assembly process associated with plant metabolic variation and soil constraints. These observations also suggest potentially promising agronomic perspectives for chicory, particularly regarding the future integration of microbiome-associated traits into breeding strategies and agroecological approaches. However, because the study was based on an observational design, genotype, developmental, seasonal, and local environmental effects could not be fully disentangled. In addition, rhizosphere physicochemical parameters and functional activities were not directly measured, and ecological interpretations were inferred from taxonomic and metabolomic associations. Consequently, further studies including controlled experiments, rhizosphere physicochemical characterization, functional assays, direct exudate analyses, larger sample sizes, and multi-site validation will be necessary to establish causal relationships and strengthen the ecological and agronomic interpretation of these findings.

## Data Availability

The sequencing datasets generated during the current study have been deposited in the NCBI repository under BioProject accession number PRJNA1441179.

## Footnotes

1
www.meteofrance.com

2
https://rast.nmpdr.org/

3
https://bacdive.dsmz.de/

4
http://fungidb.org

5
www.geoportail.gouv.fr

## References

[B1] Adhikari M. Kim S. Yadav D. Um Y. Kim H. Lee H. et al. (2016). A new record of Pseudeurotium bakeri from crop field soil in Korea. *Korean J. Mycol.* 44 145–149. 10.4489/KJM.2016.44.3.145

[B2] An D. S. Im W.-T. Yoon M.-H. (2008). Microlunatus panaciterrae sp. nov., a beta-glucosidase-producing bacterium isolated from soil in a ginseng field. *Int. J. Syst. Evol. Microbiol.* 58 2734–2738. 10.1099/ijs.0.65004-0 19060049

[B3] Anderson M. J. (2001). A new method for non-parametric multivariate analysis of variance. *Austral Ecol.* 26 32–46. 10.1111/j.1442-9993.2001.01070.pp.x

[B4] BR4 BacDive (2025). *BacDive – The Bacterial Diversity Metadatabase*. The Leibniz Institute DSMZ. Available online at: https://bacdive.dsmz.de/strain/16920 (accessed June 30, 2025).

[B5] Badri D. V. Vivanco J. M. (2009). Regulation and function of root exudates. *Plant Cell Environ.* 32 666–681. 10.1111/j.1365-3040.2009.01926.x 19143988

[B6] Bais H. P. Weir T. L. Perry L. G. Gilroy S. Vivanco J. M. (2006). The role of root exudates in rhizosphere interactions with plants and other organisms. *Annu. Rev. Plant Biol.* 57 233–266. 10.1146/annurev.arplant.57.032905.105159 16669762

[B7] Baize D. Girard M.-C. (2009). *Référentiel pédologique 2008*. Versailles: Éditions Quae. (French).

[B8] Benjamini Y. Hochberg Y. (1995). Controlling the false discovery rate: A practical and powerful approach to multiple testing. *J. R. Stat. Soc. Ser. B* 57 289–300. 10.1111/j.2517-6161.1995.tb02031.x

[B9] Bensch K. Braun U. Groenewald J. Z. Crous P. W. (2012). The genus Cladosporium. *Stud. Mycol.* 72 1–401. 10.3114/sim0003 22815589 PMC3390897

[B10] Bérdy J. (2005). Bioactive microbial metabolites. *J. Antibiotics* 58 1–26. 10.1038/ja.2005.1 15813176

[B11] Bray J. R. Curtis J. T. (1957). An ordination of the upland forest communities of southern Wisconsin. *Ecol. Monogr.* 27 325–349. 10.2307/1942268 34212107 PMC8221127

[B12] Brown C. T. Hug L. A. Thomas B. C. Sharon I. Castelle C. J. Singh A. et al. (2015). Unusual biology across a group comprising more than 15% of domain Bacteria. *Nature* 523 208–211. 10.1038/nature14486 26083755

[B13] Bulgarelli D. Schlaeppi K. Spaepen S. van Themaat E. V. L. Schulze-Lefert P. (2013). Structure and functions of the bacterial microbiota of plants. *Annu. Rev. Plant Biol.* 64 807–838. 10.1146/annurev-arplant-050312-120106 23373698

[B14] Busse H.-J. (2016). Review of the taxonomy of the genus Arthrobacter, emendation of the genus Arthrobacter sensu lato, proposal to reclassify selected species of the genus Arthrobacter in the novel genera Glutamicibacter gen. nov., Paeniglutamicibacter gen. nov., Pseudoglutamicibacter gen. nov., Paenarthrobacter gen. nov. and Pseudarthrobacter gen. nov., and emended description of Arthrobacter roseus. *Int. J. Syst. Evol. Microbiol.* 66 9–37. 10.1099/ijsem.0.000702 26486726

[B15] Castelle C. J. Banfield J. F. (2018). Biosynthetic capacity and unusual biology in the CPR radiation. *Nat. Rev. Microbiol.* 16 629–645. 10.1038/s41579-018-0076-2 30181663

[B16] Chadwick M. Trewin H. Gawthrop F. Wagstaff C. (2013). Sesquiterpenoids lactones: Benefits to plants and people. *Int. J. Mol. Sci.* 14 12780–12805. 10.3390/ijms140612780 23783276 PMC3709812

[B17] Chao A. (1984). Nonparametric estimation of the number of classes in a population. *Scand. J. Stat.* 11 265–270.

[B18] Chaparro J. M. Badri D. V. Vivanco J. M. (2014). Rhizosphere microbiome assemblage is affected by plant development. *ISME J.* 8 790–803. 10.1038/ismej.2013.196 24196324 PMC3960538

[B19] Chaverri P. Salgado C. Hirooka Y. Rossman A. Y. Samuels G. J. (2011). Delimitation of neonectria and cylindrocarpon (Nectriaceae, hypocreales, ascomycota) and related genera with cylindrocarpon-like anamorphs. *Stud. Mycol.* 68 57–78. 10.3114/sim.2011.68.03 21523189 PMC3065985

[B20] Cotrufo M. F. Lavallee J. M. Zhang Y. Hansen P. M. Paustian K. H. Schipanski M. et al. (2021). In-N-out: A hierarchical framework to understand and predict soil carbon storage and nitrogen recycling. *Glob. Change Biol.* 27 4465–4468. 10.1111/gcb.15782 34480393

[B21] de Hoog G. S. Vicente V. A. Najafzadeh M. J. Harrak M. J. Badali H. Seyedmousavi S. (2011). Waterborne Exophiala species causing disease in cold-blooded animals. *Persoonia* 27 46–72. 10.3767/003158511X614258 22403476 PMC3251318

[B22] DeAngelis K. M. Brodie E. L. DeSantis T. Z. Andersen G. L. Lindow S. E. Firestone M. K. (2009). Selective progressive response of soil microbial community to wild oat roots. *ISME J.* 3 168–178. 10.1038/ismej.2008.103 19005498

[B23] Dennis P. G. Miller A. J. Hirsch P. R. (2010). Are root exudates more important than other sources of rhizodeposits in structuring rhizosphere bacterial communities? *FEMS Microbiol. Ecol.* 72 313–327. 10.1111/j.1574-6941.2010.00860.x 20370828

[B24] FAO. (2021). *Standard Operating Procedure for Soil pH Determination*. Rome: Food and Agriculture Organization of the United Nations.

[B25] Fitzpatrick C. R. Copeland J. Wang P. W. Guttman D. S. Johnson M. T. J. (2018). Assembly and ecological function of the root microbiome across angiosperm plant species. *Proc. Natl. Acad. Sci. U S A.* 115 E1157–E1165. 10.1073/pnas.1717617115 29358405 PMC5819437

[B26] Fredrickson J. K. Balkwill D. L. Drake G. R. Romine M. F. Ringelberg D. B. White D. C. (1995). Aromatic-degrading Sphingomonas isolates from the deep subsurface. *Appl. Environ. Microbiol.* 61 1917–1922. 10.1128/aem.61.5.1917-1922.1995 7544095 PMC167454

[B27] Fukasawa Y. Osono T. Takeda H. (2011). Wood decomposing abilities of diverse lignicolous fungi on nondecayed and decayed beech wood. *Mycologia* 103 474–482. 10.3852/10-246 21262989

[B28] Gérard A. El-Hajjaji S. Burteau S. Fall P. Pirard B. Taminiau B. et al. (2021). Study of the microbial diversity of a panel of Belgian artisanal cheeses associated with challenge studies for Listeria ocytogenes. *Food Microbiol.* 100:103861. 10.1016/j.fm.2021.103861 34416961

[B29] Gibson G. R. Hutkins R. Sanders M. E. Prescott S. L. Reimer R. A. Salminen S. J. et al. (2017). The International Scientific Association for Probiotics and Prebiotics (ISAPP) consensus statement on the definition and scope of prebiotics. *Nat. Rev. Gastroenterol. Hepatol.* 14 491–502. 10.1038/nrgastro.2017.75 28611480

[B30] Gis Sol. (n.d.). *Inventaire, gestion et conservation des sols (IGCS).* Available online at: https://www.gissol.fr/le-gis/programmes/inventaire-gestion-et-conservation-des-sols-igcs-67 (accessed March 2026)

[B31] Haichar F. E. Z. Marol C. Berge O. Rangel-Castro J. I. Prosser J. I. Balesdent J. et al. (2008). Plant host habitat and root exudates shape soil bacterial community structure. *ISME J.* 2 1221–1230. 10.1038/ismej.2008.80 18754043

[B32] Häkkinen S. T. Sokoviæ M. Nohynek L. Æiriæ A. Ivanov M. Stojkoviæ D. et al. (2021). Chicory extracts and sesquiterpene lactones show potent activity against bacterial and fungal pathogens. *Pharmaceuticals* 14:941. 10.3390/ph14090941 34577641 PMC8469098

[B33] Harman G. E. Howell C. R. Viterbo A. Chet I. Lorito M. (2004). Trichoderma species—Opportunistic, avirulent plant symbionts. *Nat. Rev. Microbiol.* 2 43–56. 10.1038/nrmicro797 15035008

[B34] Hartman K. van der Heijden M. G. A. Roussely-Provent V. Walser J. C. Schlaeppi K. (2017). Deciphering composition and function of the root microbiome of a legume plant. *Microbiome* 5:2. 10.1186/s40168-016-0220-z 28095877 PMC5240445

[B35] Heiri O. Lotter A. F. Lemcke G. (2001). Loss on ignition as a method for estimating organic and carbonate content in sediments: Reproducibility and comparability of results. *J. Paleolimnol.* 25 101–110. 10.1023/A:1008119611481

[B36] Hernández-Pacheco C. E. Orozco-Mosqueda M. D. C. Flores A. Valencia-Cantero E. Santoyo G. (2021). Tissue-specific diversity of bacterial endophytes in Mexican husk tomato plants (Physalis ixocarpa Brot. ex Horm.), and screening for their multiple plant growth-promoting activities. *Curr. Res. Microb. Sci.* 2: 100028. 10.1016/j.crmicr.2021.100028 34841319 PMC8610326

[B37] Hilbert J.-L. Rambaud C. (2023). “Industrial chicory and its specialized metabolites: Diversification of uses and varietal selection,” in *Natural Products in Beverages (Reference Series in Phytochemistry*, eds Grumezescu A. M. Holban A. M. (Berlin: Springer International Publishing), 1–35. 10.1007/978-3-031-04195-2_134-1

[B38] Hopwood D. A. (2007). *Streptomyces in Nature and Medicine: The Antibiotic Makers.* Oxford: Oxford University Press.

[B39] Hosoya T. (2021). Systematics, ecology, and application of Helotiales: Recent progress and future perspectives for research with special emphasis on activities within Japan. *Mycoscience* 62 1–9. 10.47371/mycosci.2020.05.002 37090017 PMC9157779

[B40] Inderjit Duke S. O. (2003). Ecophysiological aspects of allelopathy. *Planta* 217 529–539. 10.1007/s00425-003-1054-z 12811559

[B41] IUSS WRB. (2022). *World Reference Base For Soil Resources 2022: International Soil Classification System for Naming Soils and Creating Legends for Soil Maps*, 4th Edn. Rome: International Union of Soil Sciences.

[B42] Jones D. L. Nguyen C. Finlay R. D. (2009). Carbon flow in the rhizosphere: Carbon trading at the soil–root interface. *Plant Soil* 321 5–33. 10.1007/s11104-009-9925-0

[B43] Jumpponen A. Trappe J. M. (1998). Dark septate endophytes: A review of facultative biotrophic root-colonizing fungi. *New Phytol.* 140 295–310. 10.1046/j.1469-8137.1998.00265.x 33862835

[B44] Kabata-Pendias A. (2011). *Trace Elements in Soils and Plants*, 4th Edn. Boca Raton, FL: CRC Press, 10.1201/b10158

[B45] Kageyama A. Takahashi Y. Seki T. Tomoda H. Ōmura S. (2005). Oryzihumus leptocrescens gen. nov., sp. nov. *Int. J. Syst. Evol. Microbiol.* 55 2555–2559. 10.1099/ijs.0.63799-0 16280526

[B46] Kämpfer P. Rosselló-Mora R. Scholz H. C. Welinder-Olsson C. Falsen E. Busse H.-J. (2006). Description of Pseudochrobactrum gen. nov., with the two species Pseudochrobactrum asaccharolyticum sp. nov. and Pseudochrobactrum saccharolyticum sp. nov. *Int. Int. J. Syst. Evol. Microbiol.* 56 1823–1829. 10.1099/ijs.0.64256-0 16902015

[B47] Karimi E. Aliloo A. A. Mosavi S. B. (2024). Effect of Bacillus simplex as a growth-promoting bacterium on some growth characteristics of pepper (Capsicum annuum L.) seedlings under different water conditions. *Water Soil Sci.* 34 125–141. 10.22034/ws.2024.58833.2542

[B48] Kielak A. M. Barreto C. C. Kowalchuk G. A. van Veen J. A. Kuramae E. E. (2016). The ecology of acidobacteria: Moving beyond genes and genomes. *Front. Microbiol.* 7:744. 10.3389/fmicb.2016.00744 27303369 PMC4885859

[B49] Kim J.-H. Kim W. (2016). Tumebacillus soli sp. nov., isolated from non-rhizosphere soil. *Int. J. Syst. Evol. Microbiol.* 66 2292–2296. 10.1099/ijsem.0.001009 26956136

[B50] Kirkby C. A. Kirkegaard J. A. Richardson A. E. Wade L. J. Blanchard C. Batten G. (2011). Stable soil organic matter: A comparison of C:N:P:S ratios in Australian and other world soils. *Geoderma* 163 197–208. 10.1016/j.geoderma.2011.04.010

[B51] Kulkova I. Dobrzyński J. Kowalczyk P. Bełżecki G. Kramkowski K. (2023). Plant growth promotion using Bacillus cereus. *Int. J. Mol. Sci.* 24:9759. 10.3390/ijms24119759 37298706 PMC10253305

[B52] Kwon K. K. Woo J.-H. Yang S.-H. Kang J.-H. Lee S.-T. (2007). Altererythrobacter epoxidivorans gen. nov., sp. nov., an epoxide hydrolase-active, mesophilic marine bacterium isolated from cold-seep sediment, and reclassification of Erythrobacter luteolus as Altererythrobacter luteolus comb. nov. *Int. J. Syst. Evol. Microbiol.* 57 2187–2191. 10.1099/ijs.0.64863-0 17911284

[B53] Labeda D. P. Dunlap C. A. Rong X. Huang Y. Doroghazi J. R. Ju K.-S. et al. (2017). Phylogenetic relationships in the family Streptomycetaceae using multi-locus sequence analysis. *Antonie van Leeuwenhoek* 110 563–583. 10.1007/s10482-016-0824-0 28039547 PMC10327403

[B54] Lattanzio V. Lattanzio V. M. T. Cardinali A. (2006). “Role of phenolics in the resistance mechanisms of plants against fungal pathogens and insects,” in *Phytochemistry: Advances in Research*, ed. Imperato F. Trivandrum, India: (Research Signpost), 23–67.

[B55] Leclercq L. Debarre S. Lloret E. Taminiau B. Daube G. Rambaud C. et al. (2025). Unveiling the hidden allies of industrial chicory: A metagenomic exploration of rhizosphere microbiota and their impact on productivity and plant health. *Front. Microbiol.* 16:1509094. 10.3389/fmicb.2025.1509094 40415946 PMC12098591

[B56] Liu X. Z. Wang Q. M. Göker M. Groenewald M. Kachalkin A. V. Lumbsch H. T. et al. (2015). Towards an integrated phylogenetic classification of the Tremellomycetes. *Stud. Mycol.* 81 85–147. 10.1016/j.simyco.2015.12.001 26955199 PMC4777781

[B57] Lombard L. van der Merwe N. A. Groenewald J. Z. Crous P. W. (2015). Generic concepts in nectriaceae. *Stud. Mycol.* 80 189–245. 10.1016/j.simyco.2014.12.002 26955195 PMC4779799

[B58] Loria R. Kers J. Joshi M. (2006). Evolution of plant pathogenicity in Streptomyces. *Annu. Rev. Phytopathol.* 44 469–487. 10.1146/annurev.phyto.44.032905.091147 16719719

[B59] Love M. I. Huber W. Anders S. (2014). Moderated estimation of fold change and dispersion for RNA-seq data with DESeq2. *Genome Biol.* 15:550. 10.1186/s13059-014-0550-8 25516281 PMC4302049

[B60] Lovley D. R. (1993). Dissimilatory metal reduction. *Annu. Rev. Microbiolo.* 47 263–290. 10.1146/annurev.mi.47.100193.001403 8257100

[B61] Lovley D. R. Holmes D. E. Nevin K. P. (2004). Dissimilatory Fe(III) and Mn(IV) reduction. *Adv. Microb. Physiol.* 49 219–286. 10.1016/S0065-2911(04)49005-5 15518832

[B62] Ma Y. Li X. Wang F. Zhang L. Zhou S. Che X. et al. (2023). Structural and biochemical characterization of the key components of an auxin degradation operon from the rhizosphere bacterium Variovorax. *PLoS Biol.* 21:e3002189. 10.1371/journal.pbio.3002189 37459330 PMC10374108

[B63] Madden R. H. (1983). Isolation and characterization of Clostridium stercorarium sp. nov., cellulolytic thermophile. *Int. J. Syst. Bacteriol.* 33 837–840. 10.1099/00207713-33-4-837

[B64] Marik D. Sharma P. Chauhan N. S. Jangir N. Shekhawat R. S. Verma D. et al. (2024). Peribacillus frigoritolerans T7-IITJ, a potential biofertilizer, induces plant growth-promoting genes of Arabidopsis thaliana. *J. Appl. Microbiol.* 135:lxae066. 10.1093/jambio/lxae066 38486365

[B65] McBride M. J. (2014). “The family flavobacteriaceae,” in *The Prokaryotes*, 4th Edn, eds Rosenberg E. DeLong E. F. Lory S. Stackebrandt E. Thompson F. (Berlin: Springer), 643–676. 10.1007/978-3-642-38954-2_130

[B66] Mendes R. Kruijt M. de Bruijn I. Dekkers E. van der Voort M. Schneider J. H. M. et al. (2011). Deciphering the rhizosphere microbiome for disease-suppressive bacteria. *Science* 332 1097–1100. 10.1126/science.1203980 21551032

[B67] Mountfort D. O. Rainey F. A. Burghardt J. Kaspar H. F. Stackebrandt E. (1997). Clostridium vincentii sp. nov., a new obligately anaerobic, saccharolytic, psychrophilic bacterium isolated from low-salinity pond sediment of the McMurdo Ice Shelf, Antarctica. *Arch. Microbiol.* 167 54–60. 10.1007/s002030050416 9000342

[B68] Nakamura L. K. (1998). Bacillus pseudomycoides sp. nov. *Int. J. Syst. Bacteriol.* 48 1031–1035. 10.1099/00207713-48-3-1031 9734060

[B69] Oberwinkler F. Riess K. Bauer R. Garnica S. Weiß M. (2013). Enigmatic sebacinales. *Mycol. Prog.* 12 1–27. 10.1007/s11557-012-0880-4

[B70] Ofek M. Hadar Y. Minz D. (2012). Ecology of root colonizing Massilia (Oxalobacteraceae). *PLoS One* 7:e0040117. 10.1371/journal.pone.0040117 22808103 PMC3394795

[B71] Ogaki M. B. Vieira R. Muniz M. C. Zani C. L. Alves T. M. A. Junior P. A. S. et al. (2020). Diversity, ecology, and bioprospecting of culturable fungi in lakes impacted by anthropogenic activities in Maritime Antarctica. *Extremophiles* 24 637–655. 10.1007/s00792-020-01183-z 32533308

[B72] Oksanen J. Simpson G. L. Blanchet G. Kindt R. Legendre P. Minchin P. R. et al. (2022). *vegan: Community Ecology Package (R package version x.x-x).* Available online at: https://CRAN.R-project.org/package=vegan.

[B73] Ou D. Huang H. Bai R. Li Q. Wang Y. Yin Y. (2017). Nitratireductor aestuarii sp. nov., a marine alphaproteobacterium isolated from an estuary. *Int. J. Syst. Evol. Microbiol.* 67 1722–1727. 10.1099/ijsem.0.001771 28056221

[B74] Ozimek E. Hanaka A. (2021). Mortierella species as the plant growth-promoting fungi present in the agricultural soils. *Agriculture* 11:7. 10.3390/agriculture11010007

[B75] Pagnier I. Raoult D. La Scola B. (2011). Isolation and characterization of Reyranella massiliensis gen. nov., sp. nov. from freshwater samples by using an amoeba co-culture procedure. *Int. J. Syst. Evol. Microbiol.* 61 2151–2154. 10.1099/ijs.0.025775-0 20889765

[B76] Pascale A. Proietti S. Pantelides I. S. Stringlis I. A. (2020). Modulation of the root microbiome by plant molecules: The basis for targeted disease suppression and plant growth promotion. *Front. Plant Sci.* 10:1741. 10.3389/fpls.2019.01741 32038698 PMC6992662

[B77] Philippot L. Raaijmakers J. M. Lemanceau P. van der Putten W. H. (2013). Going back to the roots: The microbial ecology of the rhizosphere. *Nat. Rev. Microbiol.* 11 789–799. 10.1038/nrmicro3109 24056930

[B78] Phillips K. E. Stevens D. C. Whitworth D. E. (2022). Concepts and conjectures concerning predatory performance of myxobacteria. *Microbiology* 168:1226. 10.1099/mic.0.001226 36246230 PMC9556981

[B79] Picman A. K. (1986). Biological activities of sesquiterpene lactones. *Biochem. Syst. Ecol.* 14, 255–281. 10.1016/0305-1978(86)90101-8

[B80] Pielou E. C. (1966). The measurement of diversity in different types of biological collections. *J. Theoretical Biol.* 13 131–144. 10.1016/0022-5193(66)90013-0

[B81] Poehlein A. Zverlov V. V. Daniel R. Schwarz W. H. Liebl W. (2013). Complete genome sequence of Clostridium stercorarium subsp. stercorarium strain DSM 8532, a thermophilic degrader of plant cell wall fibers. *Genome Announcements* 1:e00073-13. 10.1128/genomeA.00073-13 23516204 PMC3593316

[B82] Pouille C. L. Ouaza S. Roels E. Behra J. Tourret M. Molinié R. et al. (2022). Chicory: Understanding the effects and effectors of this functional food. *Nutrients* 14: 957. 10.3390/nu14050957 35267932 PMC8912540

[B83] Powell M. J. (2017). “Chytridiomycota,” in *Handbook of the Protists*, eds Archibald J. Simpson A. Slamovits C. (Berlin: Springer), 10.1007/978-3-319-28149-0_18

[B84] Proença D. N. Schwab S. Vidal M. S. Baldani J. I. Xavier G. R. Morais P. V. (2019). The nematicide Serratia plymuthica M24T3 colonizes Arabidopsis thaliana, stimulates plant growth, and presents plant beneficial potential. *Braz. J. Microbiol.* 50 777–789. 10.1007/s42770-019-00098-y 31177380 PMC6863192

[B85] Puhlmann M.-L. de Vos W. M. (2021). Back to the roots: Revisiting the use of the fiber-rich Cichorium intybus L. taproots. *Adv. Nutr.* 12 878–889. 10.1093/advances/nmaa136 34132327 PMC8321837

[B86] Reichenbach H. (2001). Myxobacteria, producers of novel bioactive substances. *J. Industrial Microbiol. Biotechnol.* 27 149–156. 10.1038/sj.jim.7000025 11780785

[B87] Roden E. E. Lovley D. R. (1993). Dissimilatory Fe(III) reduction by the marine microorganism Desulfuromonas acetoxidans. *Appl. Environ. Microbiol.* 59 734–742. 10.1128/aem.59.3.734-742.1993 16348888 PMC202183

[B88] Rognes T. Flouri T. Nichols B. Quince C. Mahé F. (2016). VSEARCH: A versatile open source tool for metagenomics. *Peer J.* 4:e2584. 10.7717/peerj.2584 27781170 PMC5075697

[B89] Rossman A. Y. Samuels G. J. Rogerson C. T. Lowen R. (1999). Genera of bionectriaceae, hypocreaceae and nectriaceae (hypocreales, ascomycetes). *Stud. Mycol.* 42 1–248.

[B90] Saini N. Gupta R. S. (2021). A robust phylogenetic framework for members of the order Legionellales and its main genera (Legionella, Aquicella, Coxiella and Rickettsiella) based on phylogenomic analyses and identification of molecular markers demarcating different clades. *Antonie van Leeuwenhoek* 114 957–982. 10.1007/s10482-021-01569-9 33881638

[B91] Saman S. Saman S. Slattery P. (2010). Isolation of a potential new member of the Bacillus cereus group from snow covered soil. *Life Sci. Med. Res.*

[B92] Santos P. Pinhal I. Rainey F. Empadinhas N. Costa J. Fields B. et al. (2003). Gamma-*proteobacteria* Aquicella lusitana gen. nov., sp. nov., and Aquicella siphonis sp. nov. infect protozoa and require activated charcoal for growth in laboratory media. *Appl. Environ. Microbiol.* 69 6533–6540. 10.1128/AEM.69.11.6533-6540.2003 14602611 PMC262295

[B93] Sasse J. Martinoia E. Northen T. (2018). Feed your friends: Do plant exudates shape the root microbiome? *Trends Plant Sci.* 23 25–41. 10.1016/j.tplants.2017.09.003 29050989

[B94] Schlaeppi K. Dombrowski N. Oter R. G. Ver Loren, van Themaat E. Schulze-Lefert P. (2014). Quantitative divergence of the bacterial root microbiota in Arabidopsis thaliana relatives. *Proc. Natl. Acad. Sci. U S A.* 111 585–592. 10.1073/pnas.1321597111 24379374 PMC3896156

[B95] Schmidt T. J. (2006). “Structure–activity relationships of sesquiterpene lactones,” in *Studies in Natural Products Chemistry*, Vol. 33 (Amsterdam: Elsevier), 309–392. 10.1016/S1572-5995(06)80030-X

[B96] Shade A. Caporaso J. G. Handelsman J. Knight R. Fierer N. (2013). A meta-analysis of changes in bacterial and archaeal communities with time. *ISME J.* 7 1493–1506. 10.1038/ismej.2013.54 23575374 PMC3721121

[B97] Shannon C. E. (1948). A mathematical theory of communication. *Bell Syst. Techn. J.* 27 379–423. 10.1002/j.1538-7305.1948.tb01338.x

[B98] Shi S. Nuccio E. E. Herman D. J. Rijkers R. Estera K. Li J. et al. (2015). Successional trajectories of rhizosphere bacterial communities over consecutive seasons. *mBio* 6:e00746-15. 10.1128/mBio.00746-15 26242625 PMC4526712

[B99] Stackebrandt E. Rainey F. A. Ward-Rainey N. L. (1997). Proposal for a new hierarchic classification system, Actinobacteria classis nov. *Int. J. Syst. Bacteriol.* 47 479–491. 10.1099/00207713-47-2-479

[B100] Sun S.-L. Yang W.-L. Fang W.-W. Zhao Y.-X. Guo L. Dai Y.-J. (2018). The plant growth-promoting rhizobacterium Variovorax boronicumulans CGMCC 4969 regulates the level of indole-3-acetic acid synthesized from indole-3-acetonitrile. *Appl. Environ. Microbiol.* 84:e00298-18. 10.1128/AEM.00298-18 29884755 PMC6070764

[B101] Świątczak J. Kalwasińska A. Brzezińska M. S. (2024). Plant growth–promoting rhizobacteria: Peribacillus frigoritolerans 2RO30 and *Pseudomonas* sivasensis 2RO45 for their effect on canola growth under controlled as well as natural conditions. *Front. Plant Sci.* 14:1233237. 10.3389/fpls.2023.1233237 38259930 PMC10800854

[B102] Takeuchi M. Hamana K. Hiraishi A. (2001). Proposal of the genus Sphingomonas sensu stricto and three new genera, Sphingobium gen. nov., Novosphingobium gen. nov. and Sphingopyxis gen. nov., on the basis of phylogenetic and chemotaxonomic analyses. *Int. J. Syst. Evol. Microbiol.* 51 1405–1417. 10.1099/00207713-51-4-1405 11491340

[B103] Thomma B. P. H. J. (2003). Alternaria spp.: From general saprophyte to specific parasite. *Mol. Plant Pathol.* 4 225–236. 10.1046/j.1364-3703.2003.00173.x 20569383

[B104] Tkacz A. Cheema J. Chandra G. Grant A. Poole P. S. (2015). Stability and succession of the rhizosphere microbiota depends upon plant type and soil composition. *ISME J.* 9 2349–2359. 10.1038/ismej.2015.41 25909975 PMC4611498

[B105] Trivedi P. Leach J. E. Tringe S. G. Sa T. Singh B. K. (2020). Plant–microbiome interactions: From community assembly to plant health. *Nat. Rev. Microbiol.* 18 607–621. 10.1038/s41579-020-0412-1 32788714

[B106] Ueda K. Yamashita A. Ishikawa J. Shimada M. Watsuji T. O. Morimura K. et al. (2004). Genome sequence of Symbiobacterium thermophilum, an uncultivable bacterium that depends on microbial commensalism. *Nucleic Acids Res.* 32 4937–4944. 10.1093/nar/gkh830 15383646 PMC519118

[B107] Uroz S. Calvaruso C. Turpault M.-P. Frey-Klett P. (2009). Mineral weathering by bacteria: Ecology, actors and mechanisms. *Trends Microbiol.* 17 378–387. 10.1016/j.tim.2009.05.004 19660952

[B108] Urzì C. Salamone P. Schumann P. Stackebrandt E. (2000). Marmoricola aurantiacus gen. nov., sp. nov., a coccoid member of the family Nocardioidaceae isolated from a marble statue. *Int. J. Syst. Evol. Microbiol.* 50 529–536. 10.1099/00207713-50-2-529 10758857

[B109] Wagner L. Stielow B. de Hoog S. Bensch K. Schwartze V. Voigt K. et al. (2013). A comprehensive molecular phylogeny of the Mortierellales. *Persoonia* 30 77–93. 10.3767/003158513X666268 24027348 PMC3734968

[B110] Wang Y. Zhang Z. Ramanan N. (1997). The actinomycete Thermobispora bispora contains two distinct types of transcriptionally active 16S rRNA genes. *J. Bacteriol.* 179 3270–3276. 10.1128/jb.179.10.3270-3276.1997 9150223 PMC179106

[B111] Wang Z. Kadouri D. E. Wu M. (2011). Genomic insights into an obligate epibiotic bacterial predator: Micavibrio aeruginosavorus ARL-13. *BMC Genomics* 12:453. 10.1186/1471-2164-12-453 21936919 PMC3189940

[B112] White D. C. Sutton S. D. Ringelberg D. B. (1996). The genus sphingomonas: Physiology and ecology. *Curr. Opin. Biotechnol.* 7 301–306. 10.1016/s0958-1669(96)80034-6 8785434

[B113] Willems A. De Ley J. Gillis M. Kersters K. (1991). Comamonadaceae, a new family encompassing the acidovorans rRNA complex, including Variovorax paradoxus gen. nov., comb. nov., for Alcaligenes paradoxus (Davis, 1969). *Int. J. Syst. Bacteriol.* 41 445–450. 10.1099/00207713-41-3-445

[B114] Yao L. Zhang J.-J. Yu L.-L. Chen Q. Zhu J.-C. He J. et al. (2016). Rhizorhabdus dicambivorans sp. nov., a dicamba-degrading bacterium isolated from compost. *Int. J. Syst. Evol. Microbiol.* 66 3317–3323. 10.1099/ijsem.0.001194 27255344

[B115] Yoon J.-H. Kang S.-J. Lee S.-Y. Lee J.-S. Park S. (2011). Ohtaekwangia koreensis gen. nov., sp. nov. and Ohtaekwangia kribbensis sp. nov., isolated from marine sand, deep-branching members of the phylum Bacteroidetes. *Int. J. Syst. Evol. Microbiol.* 61 1066–1072. 10.1099/ijs.0.025874-0 20511453

[B116] Yoon J.-H. Lee K.-C. Weiss N. Kho Y.-H. Kang K.-H. Park Y.-H. (2001). Sporosarcina aquimarina sp. nov., a bacterium isolated from seawater in Korea, and transfer of Bacillus globisporus (Larkin and Stokes 1967), Bacillus psychrophilus (Nakamura 1984) and Bacillus pasteurii (Chester 1898) to the genus Sporosarcina as Sporosarcina globispora comb. nov., Sporosarcina psychrophila comb. nov. and Sporosarcina pasteurii comb. nov., and emended description of the genus Sporosarcina. *Int. J. Syst. Evol. Microbiol.* 51 1079–1086. 10.1099/00207713-51-3-1079 11411676

[B117] Yoon J.-H. Rhee S.-K. Lee J.-S. Park Y.-H. Lee S. T. (1997). Nocardioides pyridinolyticus sp. nov., a pyridine-degrading bacterium isolated from the oxic zone of an oil shale column. *Int. J. Syst. Evol. Microbiol.* 47 933–938. 10.1099/00207713-47-4-933 9336889

[B118] Zare R. Gams W. (2001). A revision of verticillium section prostrata. IV. the genera lecanicillium and simplicillium gen. *nov. Nova Hedwigia* 73 1–50. 10.1127/nova.hedwigia/71/2001/1

[B119] Zare R. Gams W. Starink M. Summerbell R. C. (2007). Gibellulopsis, a suitable genus for Verticillium nigrescens, and Musicillium, a new genus for V. theobromae. *Nova Hedwigia* 85 463–489. 10.1127/0029-5035/2007/0085-0463

[B120] Zhalnina K. Louie K. B. Hao Z. Mansoori N. da Rocha U. N. Shi S. et al. (2018). Dynamic root exudate chemistry and microbial substrate preferences drive patterns in rhizosphere microbial community assembly. *Nat. Microbiol.* 3 470–480. 10.1038/s41564-018-0129-3 29556109

[B121] Zhou G.-C. Wang Y. Zhai S. Ge F. Liu Z.-H. Dai Y.-J. et al. (2013). Biodegradation of the neonicotinoid insecticide thiamethoxam by the nitrogen-fixing and plant-growth-promoting rhizobacterium Ensifer adhaerens strain TMX-23. *Appl. Microbiol. Biotechnol.* 97 4065–4074. 10.1007/s00253-012-4638-3 23274958

