# Supplementary material for: Root metabolite profiles support a chemical-trophic filtering hypothesis for genotype- and stage-specific rhizosphere assembly in chicory
Source: Front Microbiol. 2026 Jul 17;17:1855632. doi: 10.3389/fmicb.2026.1855632 (PMC13423886; doi:10.3389/fmicb.2026.1855632)
Supplement: Supplementary file 1 [file Table_1.docx]

**Supplementary file 1.** Technical itinerary of chicory crop.

| **Technical itinerary** | **Treatment / History** | **Period** |
| --- | --- | --- |
| **Crop history** | Wheat | March–July 2021 |
| **Winter cover** | Fallow | August 2021 – February 2022 |
| **Chemical fertilizer** | Ammonium nitrate | March 2022 |
| **Biological fertilizer** | Foliar fertilizer (Epso Top) | June 2022 |
| **Soil preparation** | Tillage | February 1, 2022 |
| **Herbicide** | Benfluralin (Bonalan) | June 14, 2022 |
|  | Triflusulfuron (Safari) | June 23, 2022 |
|  | Penoxsulam (Boa) | June 30, 2022 |
| **Fungicide** | Azoxystrobin (Ortiva Top) | August 1, 2022 |
