# Supplementary material for: Root metabolite profiles support a chemical-trophic filtering hypothesis for genotype- and stage-specific rhizosphere assembly in chicory
Source: Front Microbiol. 2026 Jul 17;17:1855632. doi: 10.3389/fmicb.2026.1855632 (PMC13423886; doi:10.3389/fmicb.2026.1855632)
Supplement: Supplementary file 2 [file Table_2.docx]

| Condition | | Sample | [DNA] ng/μL | A260/A280 | A260/A230 |
| --- | --- | --- | --- | --- | --- |
| Bulk soil | T1 | 1* | 50.6 | 1.76 | 1.1 |
|  |  | 2* | 52.6 | 1.78 | 1.1 |
|  |  | 3* | 50.8 | 1.68 | 1.23 |
|  |  | 4 | 58.6 | 1.61 | 0.71 |
|  |  | 5 | 47 | 1.58 | 1.56 |
|  | T2 | 1* | 33 | 1.59 | 1.08 |
|  |  | 2* | 44.7 | 2.8 | 1.37 |
|  |  | 3 | 32.3 | 1.34 | 1.65 |
|  |  | 4 | 38.6 | 3.88 | 1.66 |
|  |  | 5* | 41.1 | 2.32 | 1.78 |
| Far01 | T1 | 1* | 44.3 | 2.43 | 1.13 |
|  |  | 2* | 40.2 | 2.99 | 1.92 |
|  |  | 3* | 50 | 2.24 | 1.95 |
|  |  | 4 | 66.3 | 1.62 | 2.74 |
|  |  | 5 | 64.8 | 1.94 | 1.54 |
|  | T2 | 1* | 60.2 | 1.71 | 1.47 |
|  |  | 2* | 54.3 | 1.76 | 1.84 |
|  |  | 3 | 54.5 | 3.32 | 1.74 |
|  |  | 4 | 60.3 | 1.62 | 1.67 |
|  |  | 5* | 57.4 | 2.11 | 1.58 |
| Far05 | T1 | 1 | 66.2 | 1.59 | 1.68 |
|  |  | 2* | 41.3 | 2.76 | 1.46 |
|  |  | 3 | 61.3 | 1.53 | 1.1 |
|  |  | 4* | 45.7 | 2.66 | 1.73 |
|  |  | 5* | 44.3 | 2.69 | 1.18 |
|  | T2 | 1* | 59.1 | 1.84 | 1.55 |
|  |  | 2* | 53.3 | 1.67 | 1.88 |
|  |  | 3* | 45.8 | 2.11 | 2.1 |
|  |  | 4 | 44.1 | 1.42 | 1.32 |
|  |  | 5 | 32.4 | 2.65 | 0.89 |
| Far10 | T1 | 1 | 52.2 | 1.56 | 1.49 |
|  |  | 2* | 59.1 | 1.69 | 1.52 |
|  |  | 3* | 56.3 | 1.88 | 1.31 |
|  |  | 4* | 43.7 | 1.76 | 1.95 |
|  |  | 5 | 55.6 | 1.69 | 1.52 |
|  | T2 | 1 | 57.5 | 1.66 | 0.96 |
|  |  | 2 | 55.5 | 1.72 | 1.22 |
|  |  | 3* | 57 | 2.03 | 1.63 |
|  |  | 4* | 82.4 | 1.66 | 1.68 |
|  |  | 5* | 63.1 | 1.66 | 1.84 |

**Supplementary file 2. DNA quantity and quality metrics for bulk soil and chicory genotype samples (Far01, Far05, and Far10) at developmental stages T1 and T2.** Absorbance was measured at 260 nm (A260) for nucleic acids, 280 nm (A280) for proteins, and 230 nm (A230) for residual organic compounds and chemical contaminants. Replicates selected for sequencing are indicated with an asterisk.
