# Supplementary material for: Root metabolite profiles support a chemical-trophic filtering hypothesis for genotype- and stage-specific rhizosphere assembly in chicory
Source: Front Microbiol. 2026 Jul 17;17:1855632. doi: 10.3389/fmicb.2026.1855632 (PMC13423886; doi:10.3389/fmicb.2026.1855632)
Supplement: Supplementary file 3 [file Table_3.docx]

**Supplementary file 3.** **PERMANOVA analysis of genus diversity in relation to host genotype, stage of development, and its interaction.** Analysis was computed using the “vegan” package in R. Df = degrees of freedom; SS = sum of squares; Pseudo-F = F value by permutation. Statistical significance is indicated in bold (p < 0.05); p-values are based on 999 permutations.

| Bacteria (genus) | | | | | | |
| --- | --- | --- | --- | --- | --- | --- |
| **Factor** | **Df** | **SS** | **R^2^** | **F** | **p-value** | **Explained variance (%)** |
| Genotype | 2 | 1.5926 | 0.2813 | 15.72 | **0.001** | 28.13 |
| Stage | 1 | 2.1028 | 0.3715 | 41.51 | **0.001** | 37.15 |
| Genotype x Stage | 2 | 1.3577 | 0.2398 | 13.4 | **0.001** | 23.98 |
| Residual | 12 | 0.6078 | 0.1074 |  |  | 10.74 |
| Total | 17 | 5.6609 | 1.0 |  |  | 100 |
| Fungi (genus) | | | | | | |
| Genotype | 2 | 0.8700 | 0.2019 | 4.4737 | **0.001** | 20.19 |
| Stage | 1 | 1.4839 | 0.3443 | 15.2607 | **0.001** | 34.43 |
| Genotype x Stage | 2 | 0.7887 | 0.1830 | 4.0556 | **0.001** | 18.30 |
| Residual | 12 | 1.1668 | 0.2708 |  |  | 27.08 |
| Total | 17 | 4.3095 | 1.0 |  |  | 100 |
