# Supplementary material for: Root metabolite profiles support a chemical-trophic filtering hypothesis for genotype- and stage-specific rhizosphere assembly in chicory
Source: Front Microbiol. 2026 Jul 17;17:1855632. doi: 10.3389/fmicb.2026.1855632 (PMC13423886; doi:10.3389/fmicb.2026.1855632)

**Supplementary file 6.** **PCA correlation circles (variable plots) derived from UHPLC-HRMS metabolomic analysis of chicory root extracts at two developmental stages: T1 (A) and T2 (B).** Each point represents one of the 148 detected UHPLC-HRMS features. Variables highlighted in red correspond to annotated metabolite features identified based on retention time, accurate mass, elemental composition, and MS/MS fragmentation patterns. Eleven annotated variables potentially involved in genotype discrimination are indicated in red. The percentage of variance explained by each principal component is indicated on the axes.


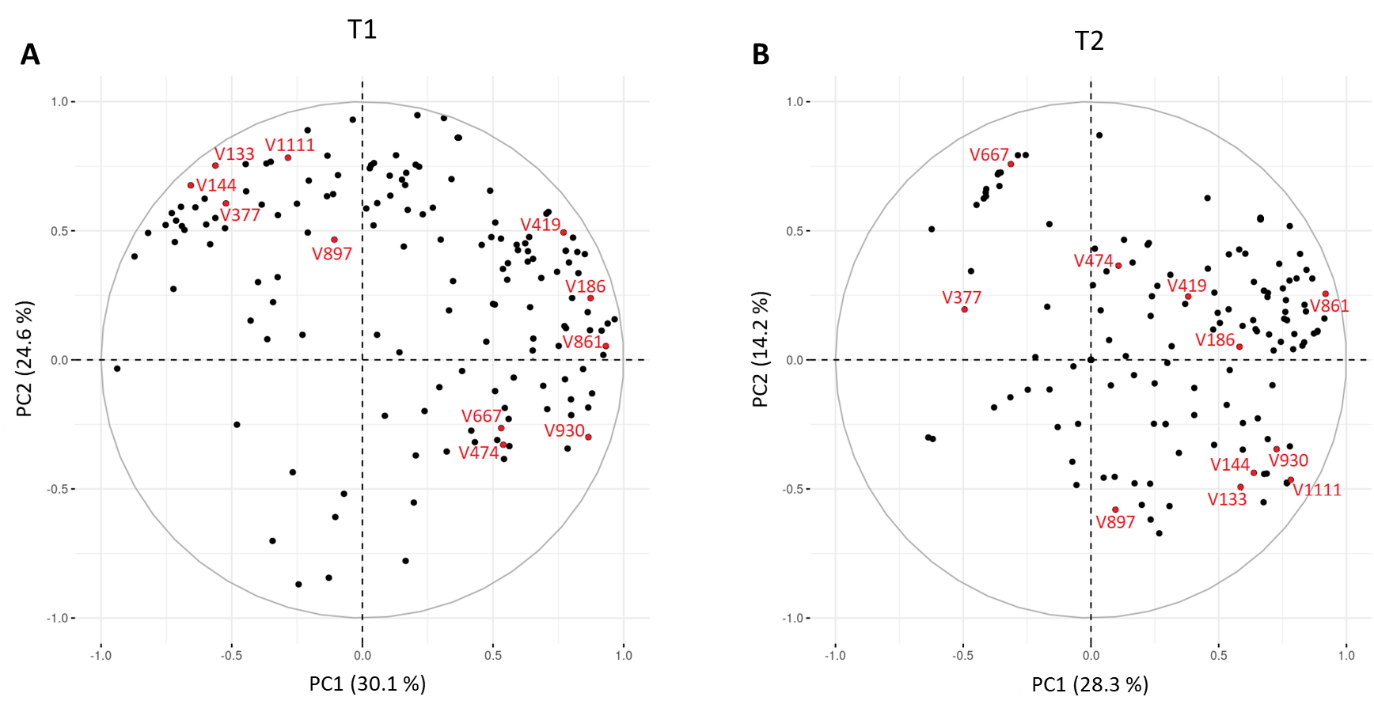

Supplement: Supplementary file 6 [file Table_6.docx]
