# Supplementary material for: Root metabolite profiles support a chemical-trophic filtering hypothesis for genotype- and stage-specific rhizosphere assembly in chicory
Source: Front Microbiol. 2026 Jul 17;17:1855632. doi: 10.3389/fmicb.2026.1855632 (PMC13423886; doi:10.3389/fmicb.2026.1855632)
Supplement: Supplementary file 7 [file Table_7.docx]

| **Kingdom** | **Stage** | **Genus** | **Metabolite** | **ρ** | **95% CI** | **p** |
| --- | --- | --- | --- | --- | --- | --- |
| Bacteria | T1 | A4b | Lactucin 15-oxalate | 0.933 | [0.58 ;1] | 0.039 |
| Bacteria | T1 | *Haliangium* | Lactucin 15-oxalate | 0.917 | [0.53 ;1] | 0.042 |
| Fungi | T1 | *Lecanicillium* | Lactucopicrin 15-oxalate | -0.950 | [-1.00 ; -0.72] | 0.007 |
| Fungi | T1 | *Spizellomycetales* | Lactucin | 0.917 | [0.48 ;1] | 0.020 |
| Fungi | T1 | *Bionectriaceae* | Lactucin | 0.883 | [0.46 ;1] | 0.031 |
| Fungi | T1 | *Bionectriaceae* | Lactucopicrin 15-oxalate | -0.883 | [-1 ; -0.51] | 0.031 |
| Fungi | T1 | *Spizellomycetales* | 11β,13-Dihydrolactucopicrin | 0.867 | [0.28 ;1] | 0.038 |
| Fungi | T1 | *Helotiales* | 11β,13-Dihydrolactucopicrin | 0.850 | [0.42 ;0.97] | 0.048 |

**Supplementary file 7. Spearman correlations between rhizosphere microbial genera and specialized metabolites.** ρ: Spearman correlation coefficient; 95% CI: bootstrap confidence intervals computed from 1,900 resamples; p: Benjamini–Hochberg FDR-adjusted p-value. Only significant correlations (adjusted p < 0.05) are shown.
